# Synthesis, Antifungal Evaluation and *In Silico* Study of *N*-(4-Halobenzyl)amides

**DOI:** 10.3390/molecules21121716

**Published:** 2016-12-13

**Authors:** Ricardo Carneiro Montes, Ana Luiza A. L. Perez, Cássio Ilan S. Medeiros, Marianna Oliveira de Araújo, Edeltrudes de Oliveira Lima, Marcus Tullius Scotti, Damião Pergentino de Sousa

**Affiliations:** 1 Departamento de Ciências Farmacêuticas, Universidade Federal da Paraíba, 58051-900 João Pessoa-PB, Brazil; ricsony_79@yahoo.com.br (R.C.M.); analuiza_perez@yahoo.com.br (A.L.A.L.P.); cassioism@hotmail.com (C.I.S.M.); marianninhaoliveira@yahoo.com.br (M.O.d.A.); edelolima@yahoo.com.br (E.d.O.L.); 2 Departamento de Química, Universidade Federal da Paraíba, 58051-900 João Pessoa-PB, Brazil; mtscotti@gmail.com

**Keywords:** halogenated amides, antimicrobial activity, *Candida*, vanillic acid derivatives

## Abstract

A collection of 32 structurally related *N*-(4-halobenzyl)amides were synthesized from cinnamic and benzoic acids through coupling reactions with 4-halobenzylamines, using (benzotriazol-1-yloxy)tris(dimethylamino)phosphonium hexafluorophosphate (BOP) as a coupling agent. The compounds were identified by spectroscopic methods such as infrared, ^1^H- and ^13^C- Nuclear Magnetic Resonance (NMR) and high-resolution mass spectrometry. The compounds were then submitted to antimicrobial tests by the minimum inhibitory concentration method (MIC) and nystatin was used as a control in the antifungal assays. The purpose of the tests was to evaluate the influence of structural changes in the cinnamic and benzoic acid substructures on the inhibitory activity against strains of *Candida albicans*, *Candida tropicalis*, and *Candida krusei*. A quantitative structure-activity relationship (QSAR) study with KNIME v. 3.1.0 and Volsurf v. 1.0.7 softwares were realized, showing that descriptors DRDRDR, DRDRAC, L4LgS, IW4 and DD2 influence the antifungal activity of the haloamides. In general, 10 benzamides revealed fungal sensitivity, especially a vanillic amide which enjoyed the lowest MIC. The results demonstrate that a hydroxyl group in the *para* position, and a methoxyl at the *meta* position enhance antifungal activity for the amide skeletal structure. In addition, the double bond as a spacer group appears to be important for the activity of amide structures.

## 1. Introduction

The genus *Candida* includes over 200 species of human pathogens. Among the most important of these are *Candida albicans*, *Candida tropicalis* and *Candida krusei*. Changes in host defense mechanisms, invasive medical procedures, and anatomical barrier failures (through burns) are all factors that favor infection with these micro-organisms [1]. However, there are compounds in Nature that can inhibit such invasions. Benzoic and cinnamic acid derivatives exhibit pharmacological versatility with antitumor, anti-inflammatory, anti-microbial and immunostimulatory activities [2,3]. In the literature, cinnamic amides inhibit the growth of fungi such as *Phytophthora infestans* [4], *Aspergillus niger*, *Candida albicans*, and bacteria such as *Escherichia coli*, *Bacillus subtilis* and *Staphylococcus aureus* [5]. Cinnamic amides with cinnamic, caffeic, ferulic, sinapic, *p*-coumaric and 3,4,5-trimethoxycinnamic cores display proven antimicrobial activity [5]. Benzoic acid acts as an unspecific antimicrobial, inhibiting β-carbonic anhydrase in *C. albicans* and *Cryptococcus neoformans* [6]. Other benzoic derivatives such as protocatequetic, gentisic, vanillic, and *p*-hydroxybenzoic acids exhibit antimicrobial properties against various bacterial and fungal strains [7].

**Figure 1:**
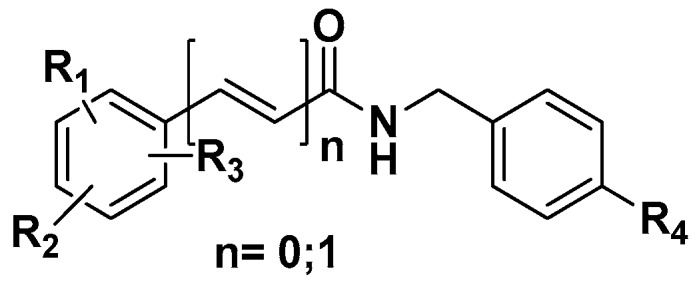
Structural skeleton of synthesized amides with substituents R_1_, R_2_ and R_3_ = H, Cl, OH, CH_3_, OMe, NO_2_, *tert*-Bu or C_6_H_5_, and R_4_ = F, Cl, or Br.

The literature reports that halogenated aromatic rings in cinnamic amides potentiate the biological activity, such as EGFR kinase inhibition [8], pesticidal effect [9,10] and microbial growth inhibition [11]. Among the halogenated amides studied, chlorinated cinnamic analogues showed higher microbial inhibition in bacterial and fungal strains than their fluorinated and brominated analogs. Furthermore, certain studies show that salicylanilides with *ortho* and *para* hydroxyls, and *meta* methoxyl on the aromatic ring all display increased microbial inhibitory activity [12]. Some studies also show the importance of substituents in the aromatic ring such as nitro groups, methyls and sterically bulky groups [13]. In this present study, we prepared a collection of structurally related cinnamic and benzoic acid amides with halogenated substituents in the *para* position, illustrated in Figure 1, in a coupling reaction using benzotriazol-1-yloxy-tris(dimethylamino) phosphonium hexafluorophosphate (BOP) as the coupling agent [14,15]. It is expected that the study will provide more information about structure-activity relationships (SAR) in this group of amides. Chemical parameters, such as lipophilicity, electronic effects, and hydrogen bonds caused by the substituents R, including the presence of the spacer (*n* = 1) between the carbonyl group and the aromatic ring, were analyzed in the SAR.

## 2. Results and Discussion

### 2.1. Chemistry

**Scheme 1:**
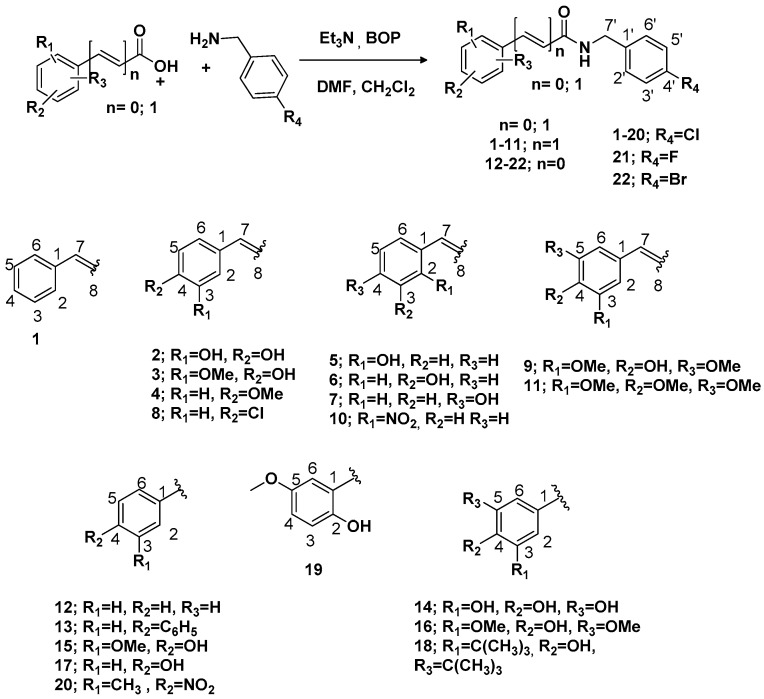
General procedure for synthesis of halogenated amides.

**Scheme 2:**
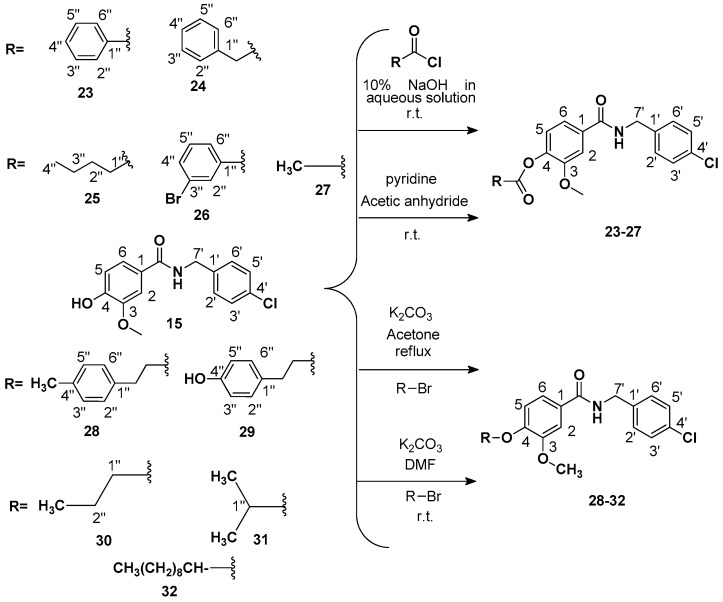
General procedure for preparation of esters and ethers **23**–**32** derived from vanillic amide (**15**). R = substituents in reactions of esters and ethers.

The structures of amides **1**–**32** (Scheme 1 and Scheme 2, Table 1 and Table 2) were consistent with the infrared spectra (IR), ^1^H- and ^13^C-NMR, and high-resolution mass spectra (MALDI) data, according to previous work [16]. The estimated purity values of the amides were at 92%–96% by ^1^H-NMR [17]. Compounds **1**–**11** were 4-chlorobenzylamides derived from cinnamic acid, and compounds **12**–**22** were *N*-(4-halobenzyl)amides derived from benzoic acid. The rings had different substituents (CH_3_, OH, OMe, F, Cl, Br, CH_3_, NO_2_, *tert-*butyl or phenyl). A vanillic acid amide had the best antifungal activity result, so five derived esters (compounds **23**–**27**) and five derived ethers (compounds **28**–**32**) of this vanillic acid amide (**15**) were prepared using reaction methods for replacing the vanillic ring OH group with an ether or ester chain. All ester and ether derivatives are reported for the first time in the literature and their spectroscopic data were in agreement with the literature data of structurally similar compounds [18,19,20,21,22,23,24,25,26].

### 2.2. Antimicrobial Activity

In the antifungal activity study, the *N*-(4-halobenzyl)amides **1**–**32** were evaluated against *Candida* strains. The technique used was the broth microdilution method according to published protocols [27,28] using seven strains of *Candida*: *C. albicans* ATCC 76645, *C. albicans* LM-106; LM-23 *C. albicans*, *C. tropicalis* ATCC 13803, *C. tropicalis*, LM-36, LM-13 *C. krusei*, and *C. krusei* LM-656. The control medium result showed no fungal growth, while growth of fungi in the medium without any added drug (sterile control) was detected.

The results are shown in Table 3; the minimum inhibitory concentrations (MICs) of the compounds were significantly different, ranging from 256 to more than 1024 μg·mL^−1^. The antimicrobial activity of the products was interpreted and considered active or not, according to the following criteria: 50–500 μg·mL^−1^ = strong/optimum activity; 600–1500 μg·mL^−1^ = moderate activity; Above 1500 μg·mL^−1^ = weak activity or inactive product [29,30]. The results of the control culture medium show no microbial growth occurred while control was positive in the yeast viability.

### 2.3. Qualitative-Structure Activity Relantioship (QSAR)

Three-dimensional structures (3D) of amides were used as input data to generate 128 descriptors together with the dependent variable (binary classification), which describes the compound as active (A) or inactive (I). The data were used as input to the KNIME v. 3.1.0 software [31]. Importantly, the generation of descriptors is relatively fast for all 32 amides which the training data sets comprised generating all 128 descriptors for Volsurf +, it took less than 1 min, using a computer equipped with an i7, running at 3.4 GHz and equipped with 12 GB of RAM. 

The KNIME software inserts the data of active and inactive compounds in mathematical algorithms that try to find the descriptors that explains the influence of the structure on microbial activity. A match is given when the software can separate the truly active and truly inactive compounds. Table 4 summarizes the statistical indices of the match model for training and cross-validation in all antifungal tests. For training set the decision tree generated high rates of correct answers for inactive compounds, up to 83.3%, and lower rates for the active compounds, 22.2%. For cross-validation, the model performed similar to the training set. The specificity (true negative) was greater than the sensitivity (true positive). Overall this means that, there was a lower false positive percentage, if it was compared with true positive prediction, which shows that the method is suitable only to screen active compounds and detect physico-chemical properties of inactive compounds.

According to the decision tree (an organization chart that compares the descriptors, to predict the biological activity), the prediction of actives and inactives was based on two Volsurf descriptors [32] of *N*-(4-halobenzyl)amides: DRDRDR and DRDRAC, which are pharmacophore descriptors forming the maximum triangular area DRY-DRY-DRY (three hydrophobic regions), and DRY-DRY-Acceptor (two hydrophobic regions and one H-bond acceptor region).

**Figure 2:**
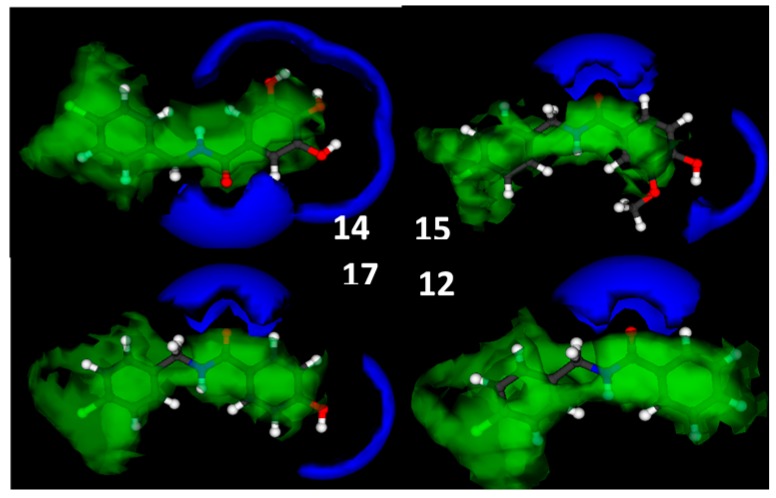
Gallic amide (**14**), vanillic amide (**15**), 4-hydroxybenzoic amide (**17**) and benzoic amide (**12**) interaction fields: energy level −0.6 kcal/mol DRY, and −3 kcal/mol N1 in VolSurf.

It was possible to establish regions for analogs that produced fungal growth sensitivity. Figure 2 shows the molecular interaction of the grid fields (Molecule Interaction Fields—MIF) around the most active compounds **14** and **15**, the weakly active compound **17** and inactive compound **12**. The DRY probe is shown in all active and weakly active structures. Looking at the N1 probe (dark blue), regions in the aromatic ring which show an outstanding hydrogen accepting character could be observed. This N1 probe is seen more present near the gallic (**14**), vanillic (**15**), and 4-hydroxybenzoic (**17**) amide hydroxyls. This study did not have a high accuracy rate for matches, decreasing the specificity of software to find descriptors related to biological activity of the evaluated amides.

## 3. Materials and Methods

### 3.1. General Information

Purification of the compounds was performed by column chromatography on silica gel 60 (ART. 7734 Merck, Saint Louis, MO, USA) using a Hex:EtOAc solvent gradient and confirmed by analytical thin layer chromatography on silica gel 60 F_254_, using ultraviolet light at two wavelengths (254 and 366 nm) from a Mineralight apparatus (UVP, Upland, CA, USA) or H_2_SO_4_ in 5% ethanol for detection. FTIR spectra were recorded in a Prestige-21 FTIR spectrometer (Shimadzu, Kyoto, Japan) using KBr pellets. ^1^H- and ^13^C-NMR spectra were obtained on a MERCURY machine (200 and 50 MHz for ^1^H and ^13^C, respectively. Varian (Palo Alto, CA, USA) in deuterated solvents (CDCl_3_, MeOD or DMSO-*d*_6_) and tetramethylsilane (TMS) was used for the internal standard. Chemical shifts were measured in parts per million (ppm) and coupling constants (*J*) in Hz. Measurements of atomic mass for the compounds was carried out using an Ultraflex II TOF/TOF mass spectrometer (Bruker Daltonik GmbH, Bremen, Germany) equipped with a high performance solid state laser (λ = 355 nm), and reflector. The system was operated by the Bruker Daltonik FlexControl 2.4 software package (Bruker, Bremen, Germany). Infrared spectrum, ^1^H-NMR spectrum, Expansion of the spectrum of ^1^H-NMR, ^13^C-APT NMR spectrum, expansion of the ^13^C-APT NMR spectrum, High resolution mass spectrum—MALDI of **2**, **3**, **14**, **16**, **23** and **28** are available at the Supplementary Materials.

### 3.2. Chemistry: General Procedures for the Preparation of Compounds

#### 3.2.1. General Preparation of *N*-(4-Halobenzyl)amides

Procedure 1: In a 100 mL flask equipped with magnetic stirring, the organic acid (1.35 mmol, 200 mg) was dissolved in dimethylformamide (DMF, 2.7 mL) and trimethylamine (0.14 mL, 1.35 mmol). The solution was cooled in an ice bath (0 °C). Then, 4-chlorobenzylamine (1.35 mmol) was added. Soon after a 1.35 mmol solution of BOP in CH_2_Cl_2_ (10 mL) was added to the flask. The reaction was stirred at 0 °C for 30 min, and then for an additional period, at room temperature for 2 h. After the reaction, the CH_2_Cl_2_ was removed under reduced pressure and the solution was poured into a separatory funnel containing water (10 mL) and EtOAc (10 mL). The product was extracted with EtOAc (3 × 10 mL). The organic phase was washed sequentially with 1 N HCl, water, 1 M NaHCO_3_ and water (10 mL of each); dried with Na_2_SO_4_, filtered and concentrated in a rotavapor. The amide was purified by gel chromatography on a silica gel column using as the mobile phase an EtOAc:Hex mixture gradient of increasing polarity [11]. The following compounds were prepared by this procedure:

*N-(4-Chlorobenzyl)cinnamamide* (**1**). Crystalline solid; 75% yield (274 mg), m.p.: 152–156 °C, IR ν_max_ (cm^−1^): 3253 (N-H), 3080 (CH sp^2^), 1654 (C=O), 1616 and 1489 (aromatic C=C), 1040 (stretching C-Cl), ^1^H-NMR (DMSO-*d*_6_): 7.71 (d, *J =* 16 Hz, 1H, H-7); 7.55–7.50 (m, 2H, H-2, H-6); 7.44–7.35 (m, 3H, H-3); 7.32–7.29 (m, 4H, H-2′, H-3′, H-5′, H-6′); 6.45 (d, *J =* 16 Hz, 1H, H-8); 6.03 (bs, 1H, O=C-NH); 4.57 (d, *J =* 4.0 Hz, H-7′). ^13^C-NMR (DMSO-*d*_6_) 166.0 (C=O); 141.9 (C-7); 136.9 (C-1′); 134.8 (C-1); 133.5 (C-4′); 130.0 (C-2′, C-6′); 129.4 (C-3′, C-5′); 129.0 (C-3, C-5, C-6); 128.0 (C-2, C-4); 120.2 (C-8); 43.3 (C-7′) [16].

*(E)-N-(4-Chlorobenzyl)-3-(3,4-dihydroxyphenyl)acrylamide* (**2**). Dark amorphous solid; yield: 70% (228 mg), m.p.: 103–105 °C, IR ν_max_ (cm^−1^): 3460 and 3406 (OH) 3230 (NH), 3018 (CH sp^2^), 1651 (C=O) 1602 and 1490 (aromatic C=C), 1089 (C-Cl stretch). ^1^H-NMR (CD_3_OD): 7.55 (d, *J =* 15.7 Hz, 1H; H-7), 7.44–7.38 (m, 4H; H-2′, H-3′, H-5′, H-6′); 7.13 (d, *J =* 1.7 Hz, 1H; H-2); 7.02 (dd, *J =* 8.2, 1.8 Hz, 1H; H-5), 6.88 (d, *J =* 8.1 Hz, 1H; H-6), 6.52 (d, *J =* 15.7 Hz, 1H, H-8), 4.55 (bs, 2H; H-7′). ^13^C-NMR (CD_3_OD): 169.2 (C=O); 148.8 (C-4); 146.7 (C-3); 142.8 (C-7); 138.9 (C-1′); 131,4 (C-4′); 130.1 (C-2′, C-6′); 129.6 (C-3′; C-5′); 128.2 (C-1); 122.2 (C-6); 118.0 (C-8); 115.0 (C-2); 116.4 (C-5); 43.5 (C-7′) [16]. HRMS (MALDI) calculated for C_16_H_14_ClNNaO_3_ [M + Na]^+^: 326.0560; found 326.0561.

(*E)-N-(4-Chlorobenzyl)-3-(4-hydroxy-3-methoxyphenyl)acrylamide* (**3**). White amorphous solid; yield: 81% (273 mg), m.p. 129–131 °C, IR ν_max_ (cm^−1^): 3414 (OH) 3275 (NH), 3010 (CH sp^2^), 1645 (C=O), 1612 and 1460 (aromatic C=C), 1039 (C-Cl stretch). ^1^H-NMR (DMSO-*d*_6_): 7.59 (d, *J =* 16 Hz, 1H; H-7) 7.28 (dd, *J =* 2.0 Hz and 6.0 Hz, 2H; H-2′, H-6′); 7.22 (dd, *J =* 2.0 Hz, 6.0 Hz, 2H, H-3′, H-5′) 7.13 (s, 1H; H-2); 6.95 (d, *J =* 8.1 Hz; 1H; H-5); 6.87 (d, *J =* 8.1 Hz; 1H; H-6); 6.27(d, *J =* 16 Hz, 1H; H-8); 4.36 (d, *J =* 6.0 Hz, 2H, H-7′); 3.79 (*s*, 3H, OCH_3_). ^13^C-NMR (DMSO-*d*_6_): 165.5 (C=O), 148.4 (C-3), 147.8 (C-4), 139.6 (C-7), 138.7 (C-1′), 131.3 (C-4′), 129.2 (C-2′, C-6′), 128.3 (C-3′, C-5′), 126.3 (C-1), 121.7 (C-6), 118.6 (C-8), 115.7 (C-5), 110.8 (C-2), 55.6 (OCH_3_), 41.6 (C-7′) [16]. HRMS (MALDI) calculated for C_17_H_16_ClNO_3_ [M + H]^+^: 318.0887; found 318.0870.

*(E)-N-(4-Chlorobenzyl)-3-(4-methoxyphenyl)acrylamide* (**4**). Crystalline solid; yield: 91% (311 mg), m.p. 148–150 °C, IR ν_max_ (cm^−1^): 3282 (N-H), 3041 (C-H sp^2^), 1647 (C=O), 1602 and 1462 (aromatic C=C), 1029 (stretching C-Cl). ^1^H-NMR (DMSO-*d*_6_): 8.59 (t, *J =* 6.0 Hz, 1H, O=C-NH), 7.50 (t, *J =* 7.3 Hz, 2H; H-2; H-6), 7.44–7.18 (m, 5H; H-7, H-2′, H-3′, H-5′, H-6′), 6.97 (d, *J =* 8.7 Hz, 2H, H-3, H-5), 6.54 (d, *J =* 15.7 Hz, 1H; H-8), 4.38 (d, *J =* 6.0 Hz, 2H; H-7′), 3.77 (s, 3H, OCH_3_). ^13^C-NMR (DMSO-*d*_6_): 165.5 (C=O); 160.4 (C-4); 139.0 (C-7); 138.7 (C-1′); 131.4 (C-4′); 129.3 (C-2; C-6, C-2′; C-6′); 128.3 (C-3′, C-5′); 127.4 (C-1); 119.4 (C-8); 114.5 (C-3, C-5); 55.3 (OCH_3_); 41.7 (C-7′) [16]. HRMS (MALDI) calculated for C_16_H_14_ClNO_2_ [M + H]^+^: 324.0767; found 324.0771.

*(E)-N-(4-Chlorobenzyl)-3-(2-hydroxyphenyl)acrylamide* (**5**). Yellow amorphous solid; yield: 79% (277 mg), m.p.: 165–171 °C. ν_max_ IR (cm^−1^): 3369 (OH), 3072 (C-H sp^2^), 1649 (C=O), 1589 and 1458 (aromatic C=C), 1093 (C-Cl stretch). ^1^H-NMR (DMSO-*d*_6_) 10.00 (bs, 1H, OH), 8.64 (t, *J =* 5.83 Hz, 1H; O=C-NH), 7.78 (d, *J =* 15.92 Hz; 1H; H-7), 7.49–7.15 (m, 6H; H-6, H-2′, H-3′, H-5′, H-6′), 6.91–6.76 (m, 2H; H-4, H-5), 6.73 (d, *J =* 15.92 Hz, 1H; H-8), 4.47 (d, *J =* 5.86 Hz, 2H; H-7′). ^13^C-NMR (DMSO-*d*_6_) 165.8 (C=O); 156.4 (C-2); 138.8 (C-1′); 135.2 (C-4); 135.0; 130.6 (C-4′); 129.3(C-2′, C-6′); 128.4 (C-6); 128.3 (C-3′, C-5′); 121.6 (C-1); 121.3 (C-5); 119.4 (C-8); 116.2; 41.7 (C-7′) [16]. HRMS (MALDI) calculated for C_16_H_14_ClNO_2_ [M + H]^+^: 288.0781; found 288.0785.

(*E)-N-(4-Chlorobenzyl)-3-(3-hydroxyphenyl)acrylamide* (**6**). Yellow crystalline solid; yield: 76% (268 mg), m.p.: 140–143 °C ν_max_ IR (cm^−1^): 3460 (OH), 3075 (CH sp^2^), 1649 (C=O), 1591 and 1448 (aromatic C=C), 1089 (C-Cl stretch). ^1^H-NMR (DMSO-*d*_6_): 9.61 (s, 1H, OH), 8.67 (t, *J =* 5.9 Hz, 1H, O=C-NH), 7.47–7.14 (m, 6H; H-5, H-7, H-2′, H-3′, H-5′ e H-6′), 7.03–6.92 (m, 2H; H-4, H-6), 6.84–6.70 (m, 1H; H-2), 6.60 (d, *J =* 15.8 Hz, 1H; H-8), 4.38 (d, *J =* 5.9 Hz, 2H; H-7′). ^13^C-NMR (DMSO-*d*_6_): 165.1 (C=O); 157.7 (C-3); 139.4 (C-7); 138.5 (C-1′); 136.1 (C-1); 131.4 (C-4′); 130.0 (C-5); 129.3 (C-2′, C-6′); 128.3 (C-3′, C-5′); 121.7 (C-8); 118.8 (C-6); 116.8 (C-2); 113.8 (C-4); 41.7 (C-7′) [16]. HRMS (MALDI) calculated for C_16_H_14_ClNNaO_2_ [M + Na]^+^: 310.0611; found 310.0619.

*(E)-N-(4-Chlorobenzyl)-3-(4-hydroxyphenyl)acrylamide* (**7**). Crystalline solid; yield: 63% (221 mg), m.p. 157–160 °C. IV ν_max_ (cm^−1^): 3479 and 3369 (OH), 3072 (C-H sp^2^), 1647 (C=O), 1589 and 1458 (aromatic C=C), 1093 (stretching C-Cl). ^1^H-NMR (DMSO-*d*_6_): 9.87 (bs, 1H, OH); 8.53 (t, *J =* 5.87 Hz, 1H, O=C-NH); 7.45–7.36 (m, 5H, H-2, H-6, H-7, H-2′, H-6′); 7.35–7.25 (m, 3H; H-2; H-3′e H-5′); 6.77 (d, *J =* 8.5 Hz, 2H; H-3 e H-5); 6.44 (d, *J =* 15.73 Hz, 1H; H-8); 4.36 (d, *J* = 6.0 Hz, 2H; H-7′). ^13^C-NMR (DMSO-*d*_6_) 165.6 (C=O); 159.0 (C-4); 139.4 (C-7); 138.8 (C-1′); 131.4 (C-4′); 129.4 (C-2, C-6); 129.3 (C-2′, C-6′); 128.3 (C-3′, C-5′); 125.9 (C-1); 118.3 (C-8); 115.8 (C-3, C-5); 41.6 (C-7′) [16]. HRMS (MALDI) calculated for C_16_H_14_ClNNaO_2_ [M + Na]^+^: 310.0611; found 310.0596.

*(E)-N-(4-Chlorobenzyl)-3-(4-chlorophenyl) acrylamide* (**8**). Crystalline solid; yield: 71% (240 mg), m.p. 157–161 °C. IV ν_max_ (cm^−1^): 3414 (N-H), 3041 (C-H sp^2^), 1651 (C=O), 1614 and 1487 (aromatic C=C), 1089 (stretching C-Cl). ^1^H-NMR (DMSO-*d*_6_): 8.70 (t, *J =* 6.0 Hz, 1H, O=C-NH), 7.64–7.46 (m, 4H, H-2′, H-3′, H-5′, H-6′), 7.45–7.25 (m, 5H; H-2, H-6, H-3, H-5, H-7), 6.69 (d, *J =* 15.8 Hz, 1H; H-8), 4.39 (d, *J =* 6.0 Hz, 2H). ^13^C-NMR (DMSO-*d*_6_) 164.9 (C=O); 138.5 (C-1′); 137.9 (C-7); 134.0 (C-4); 133.8 (C-1); 131.5 (C-4′); 129.3 (C-2, C-6, C-2′ e C-6′); 129.0 (C-3, C-5); 128.3 (C-3′, C-5′);122.7 (C-8); 41.7 (C-7′) [16]. HRMS (MALDI) calculated for C_16_H_13_Cl_2_NNaO [M + Na]^+^: 328.0272; found 328.0273.

*(E)-N-(4-Chlorobenzyl)-3-(4-hydroxy-3,5-dimethoxyphenyl)-acrylamide* (**9**). Yellow amorphous solid; yield: 60% (193 mg), m.p.: 182–185 °C, IR ν_max_ (cm^−1^): 3414 (OH) and 3358 or(NH), 3000 (CH sp^2^), 1658 (C=O), 1624 and 1458 (aromatic C=C), 1091 (C-Cl stretch). ^1^H-NMR (DMSO-*d*_6_) 8.52 (t, *J =* 6.0 Hz; 1H, O=C-N**H**), 7.28–7.48 (m, 5H; H-7, H-2′, H-3′, H-5′, H-6′), 6.86 (s, 2H; H-2, H-6), 6.55 (d, *J =* 15.8, 1H; H-8), 4.37 (d, *J =* 6 Hz, 2H; H-7′) 3.79 (s, 6H; OCH_3_). ^13^C-NMR (DMSO-*d*_6_) 165.6 (C=O); 148.1 (C-3, C-5); 140.0 (C-7) 138.7 (C-4); 137.4 (C-1′); 131.4; C-4′); 129.2 (C-2′, C-6′); 128.4 (C-3′, C-5′); 125.3 (C-1); 119.1 (C-8); 105.3 (C-2, C-6); 56.0 (OCH_3_); 41.7 (C-7′) [16]. HRMS (MALDI) calculated for C_18_H_18_ClNNaO_4_ [M + Na]^+^: 370.0822; found 370.0813.

*(E)-N-(4-Chlorobenzyl)-3-(2-nitrophenyl)-acrylamide* (**10**). White crystalline solid; yield: 79% (260 mg), m.p.: 164–167 °C, IR ν_max_ (cm^−1^): 3290 (NH), 3030 (CH sp^2^), 1651 (C=O), 1624 and 1458 (aromatic C=C), 1525 and 1342 (C=O), 1091 (C-Cl stretch). ^1^H-NMR (DMSO-*d*_6_): 8.82 (t, *J =* 4.7 Hz, 1H, O=C-NH), 8.05 (d, *J =* 8.0 Hz, 1H; H-3), 7.78–7.75 (m, 2H; H-6, H-7), 7.72–7.57 (*m*, 2H; H-4, H-5), 7.43–7.27 (m, 4H, H-2′, H-3′, H-5′ e H-6′), 6.67 (d, *J =* 5.6 Hz, 1H; H-8), 4.39 (d, *J =* 5.9 Hz, 1H; H-7′). ^13^C-NMR (DMSO-*d*_6_): 164.3 (C=O); 148.4 (C-2); 138.3 (C-1′); 134.3 (C-7); 133.9 (C-5); 131.6 (C-4′); 130.4 (C-4); 130.0 (C-1); 129.4 (C-2′, C-6′); 128.8 (C-6); 128.4 (C-3′, C-5′); 126.6 (C-3); 124.7 (C-8); 41.8 (C-7′) [16]. HRMS (MALDI) calculated for C_16_H_13_ClN_2_O_3_ [M + H]^+^: 317.0683; found 317.0683.

*(E)-N-(4-Chlorobenzyl)-3-(3,4,5-trimethoxyphenyl) acrylamide* (**11**). Crystalline solid; yield: 86% (260 mg), m.p. 146–150 °C, IR ν_max_ (KBr, cm^−1^): 3290 (N-H), 3070 (C-H sp^2^), 1651 (C=O), 1614 and 1415 (aromatic C=C), 1029 (stretching C-Cl). ^1^H-NMR (DMSO-*d*_6_): 8.60 (t, *J =* 5.9 Hz, 1H; O=C-NH), 7.46–7.26 (m, 5H; H-7, H-2′, H-3′, H-5′, H-6′), 6.90 (s, 2H; H-2, H-6), 6.64 (d, *J =* 15.7 Hz, 1H; H-8), 4.38 (d, *J =* 5.9 Hz, 1H; H-7′), 3.80 (s, 6H; *m*-OCH**_3_**). 3.68 (s, 3H; *p*-OCH**_3_**). ^13^C-NMR (DMSO-*d*_6_) 165.2 (C=O); 153.1 (C-3, C-5); 139.4 (C-7); 138.7 (C-4); 138.6 (C-1′); 131.4 (C-4′); 130.5 (C-1); 129.2 (C-2′, C-6′); 128.4 (C-3′, C-5′); 121.3 (C-8); 105.1 (C-2, C-6); 60.2 (C-4-OCH_3_); 55.9 (C-3,5-OCH_3_); 41.7 (C-7′) [16]. HRMS (MALDI) calculated for C_19_H_20_ClNO_4_ ([M + H]^+^: 384.0979, found 384.0913.

*N-(4-Chlorobenzyl) benzamide* (**12**). Crystalline solid; yield: 65% (260 mg), m.p. 136–139 °C, IR ν_max_ (cm^−1^): 3300 (N-H), 3082 (C-H sp^2^), 1637 (C=O), 1618 and 1490 (aromatic C=C), 1091 (stretching C-Cl). ^1^H-NMR (DMSO-*d*_6_): 9.10 (t, *J =* 5.9 Hz, 1H, O=C-NH), 7.89 (dd, *J =* 8.0 e 1.6 Hz, 2H; H-2, H-6), 7.64–7.09 (m, 7H; H-3, H-4, H-5, H-2′, H-3′, H-5′, H-6′), 4.46 (d, *J =* 6.0 Hz, 2H; H-7′). ^13^C-NMR (DMSO-*d*_6_): 166.4 (C=O); 138.8 (C-1′); 134.2 (C-1); 131.4 (C-4); 131.3 (C-2′, C-6′); 129.1 (C-3′, C-5′); 128.3 (C-3, C-5); 127.3 (C-2, C-6); 42.1 (C-7′) [16].

*N-(4-Chlorobenzyl)-*[1,1′-biphenyl]*-4-carboxamide* (**13**). The product was prepared according to procedure 1. White amorphous solid; yield: 56% (198 mg), m.p. 222–228 °C, IR ν_max_ (cm^−1^): 3271 (N-H), 3078 (C-H sp^2^), 1633 (C=O), 1606 and 1487 (aromatic C=C), 1089 (stretching C-Cl). ^1^H-NMR (DMSO-*d*_6_): 9.15 (t, *J =* 6.1 Hz, 1H, O=C-NH), 7.99 (d, *J =* 8.3 Hz, 2H; H-2, H-6), 7.84–7.65 (m, 4H; H-3, H-5, H-2″, H-6″), 7.56–7.29 (m, 7H; H-2′, H-3′, H-5′, H-6′, H-3″ H-4″, H-5″), 4.48 (d, *J =* 5.9 Hz, 2H). ^13^C-NMR (DMSO-*d*_6_) 166.1 (C=O); 143.0 (C-4); 139.2 (C-1″); 138.8 (C-1′); 133.0 (C-1); 131.4 (C-4′); 129.2 (C-2′, C-6′); 128.4 (C-2, C-6); 128.1 (C-9, C-11); 127.0 (C-3′, C-5′); 126.7 (C-10) (C-3, C-5, C-8, C-12); 42.1 (C-7′) [16]. HRMS (MALDI) calculated for C_20_H_16_ClNO_3_ [M + H]^+^: 322.0988; found 322.0969.

*N-(4-Chlorobenzyl)-3,4,5-trihydroxybenzamide* (**14**). Yellow amorphous solid; yield: 21% (73 mg), m.p. 96–100 °C, IR ν_max_ (cm^−1^): 3400 (OH), 3400 (NH), 3000 (CH sp^2^), 1614 (C=O), 1589 and 1494 (aromatic C=C), 1043 (stretching C-Cl). ^1^H-NMR (MeOD): 8.12 (s, 1H; O=C-NH), 7.53–7.15 (m, 4H, H-2′, H-3′, H-5′, H-6), 6.85 (s, 2H; H-2, H-6), 4.58 (s, 2H, H-7′), 4.46 (s, 2H; *m*-OH), 4.35 (s, 1H, *p*-OH). ^13^C-NMR (MeOD): 170.5 (C=O), 146.7 (C-3, C-5), 139.4 (C-4), 136.1 (C-1), 134.2 (C-4), 130.5 (C-5′, C-6′), 130.1 (C-3′, C-5′), 125.9 (C-1), 107.8 (C-2, C-6), 43.6 (C-7′) [16]. HRMS (MALDI) calculated for C_16_H_12_ClNNaO_3_ [M + Na]^+^: 316.0353; found 316.0373.

*N-(4-Chlorobenzyl)-4-hydroxy-3-methoxybenzamide* (**15**). White amorphous solid; yield: 44% (151 mg), m.p.: 75–77 °C, IR ν_max_ (cm^−1^): 3319 (O-H) 3251 (N-H), 3000 (C-H sp^2^), 1639 (C=O), 1589 and 1487 (aromatic C=C), 1091 (stretching C-Cl). ^1^H-NMR (DMSO-*d*_6_): 8.12 (d, *J =* 9.2 Hz, 1H; O=C-NH), 7.56–7.22 (m, 7H; H-2, H-4, H-6, H-3′, H-5′, H-2′, H-6′), 4.52 (s, 2H), 3.88 (s, 1H; OCH**_3_**). ^13^C-NMR (DMSO-*d*_6_): 169.8 (C=O); 151.3 (C-3); 148.8 (C-4); 139.3 (C-1′); 133.8 (C-4′); 130.1 (C-2′, C-6′); 129.5 (C-5′, C-3′); 126.5 (C-1); 122.1 (C-5); 115.8 (C-6); 111.9 (C-2); 56.4 (3-OMe); 43.8 (C-7′) [16]. HRMS (MALDI) calculated for C_16_H_16_ClNO_4_ [M + Na]^+^: 316.0530; found 316.0543.

*N-(4-Chlorobenzyl)-4-hydroxy-3,5-dimethoxybenzamide* (**16**). White amorphous solid; yield: 50% (164 mg), m.p. 110–115 °C, IR ν_max_ (KBr, cm^−1^): 3493 (OH), 3277 (NH), 3084 (CH sp^2^), 1666 (C=O), 1597 and 1492 (aromatic C=C), 1016 (stretching C-Cl). ^1^H-NMR (MeOD): 8.15 (s, 1H, O=C-NH), 7.43 (s, 1H, OH), 7.35–7.27 (m, 4H; H-2′, H-3′, H-5′, H-6′), 7.25–7.15 (m, 2H; H-2, H-6), 4.54 (s, 2H, H-7′), 3.88 (*s*, 6H; OCH_3_). ^13^C-NMR (MeOD): 149.0 (C-3, C-5), 169.8 (C=O), 139.3 (C-4), 133.8 (C-1′), 130.1 (C-2′, C-6′), 129.5 (C-3′, C-5′), 125.3 (C-4′), 106.4 (C-1), 106.0 (C-2, C-6), 43.9 (C-7′), 56.8 (OCH_3_) [16]. HRMS (MALDI) calculated for C_16_H_16_ClNNaO_4_ [M + Na]^+^: 346.0636; found 346.0666.

*N-(4-Chlorobenzyl)-4-hydroxybenzamide* (**17**). White amorphous solid; yield: 73% (246 mg), m.p. 189–192 °C, IR ν_max_ (cm^−1^): 3450 (OH), 3122 (NH), 3055 (CH sp^2^), 1631 (C=O), 1593 and 1440 (aromatic C=C), 1085 (stretching C-Cl). ^1^H-NMR (DMSO-*d*_6_): 8.83 (t, *J =* 5.9 Hz, 1H; O=C-NH), 7.75 (d, *J =* 8.6 Hz, 2H, H-2, H-6), 7.59–7.43 (m, 4H, H-2′; H-3′, H-5′, H-6′), 6.80 (d, *J =* 8.8 Hz, 2H, H-3, H-5), 4.42 (d, *J =* 6.0 Hz, 2H; H-7′). ^13^C-NMR (DMSO-*d*_6_): 166.0 (C=O); 160.3 (C-4); 139.2 (C-1′); 133.4 (C-4′); 130.9 (C-2, C-6); 129.3 (C-2′, C-6′); 128.3 (C-3′, C-5′); 124.9 (C-1); 114.9 (C-3, C-5); 41.7 (C-7′) [16]. HRMS (MALDI) calculated for C_14_H_12_ClNNaO_2_ [M + Na]^+^: 286.0425; found 286.0432.

*3,5-Di-tert-butyl-N-(4-chlorobenzyl)-4-hydroxybenzamide* (**18**). White amorphous solid; yield: 54% (203 mg), m.p. 184–186 °C, IR ν_max_ (cm^−1^): 3450 (OH) and 3236 (NH), 3066 (CH sp^2^), 1680 (C=O), 1544 and 1431 (aromatic C=C), 1012 (stretching C-Cl). ^1^H-NMR (DMSO-*d*_6_): 8.89 (t, *J =* 6.0 Hz, 1H; O=C-NH), 6.09–5.94 (m, 1H), 7.70–7.54 (m, 2H, H-2, H-6), 7.54–7.20 (m, 4H, H-2′, H-3′, H-5′, H-6′), 4.42 (d, *J =* 5.9 Hz, 3H; H-7′), 1.39 (s, 18H; C(CH**_3_**)_3_). ^13^C-NMR (DMSO-*d*_6_): 167.0 (C=O); 156.9 (C-4); 140.0 (C-1′); 138.3 (C-3, C-5); 131.2 (C-4′); 129.2 (C-2′, C-6′); 128.3 (C-5′, C-3′); 125.3 (C-1); 124.2 (C-2, C-6); 42.0 (C-7′); 34.7 (3,5-(**C**(CH_3_)_3_; 30.3 (3,5-(C(**C**H_3_)_3_) [16] HRMS (MALDI) calculated for C_22_H_28_ClNO_3_ [M + H]^+^: 374.1877; found 374.1877.

*N-(4-Chlorobenzil)-2-hydroxy-5-methoxybenzamide* (**19**). Crystalline solid; yield: 60% (180 mg), m.p. 137–140 °C, IR ν_max_ (cm^−1^): 3360 (O-H and N-H), 3076 (C-H sp^2^), 1651 (C=O), 1598 and 1435 (aromatic C=C), 1045 (stretching C-Cl). ^1^H-NMR (DMSO-*d*_6_): 9.35 (t, *J =* 5.8 Hz, 1H, O=C-NH), 7.48–7.26 (m, 5H, H-6, H-2′, H-3′, H-5′, H-6′), 7.04 (dd, *J =* 9.0, 3.0 Hz, 1H; H-3), 6.85 (d, *J =* 9.0 Hz, 1H; H-4), 4.49 (d, *J =* 5.9 Hz, 2H, H-7′), 3.72 (s, 3H; OCH**_3_**). ^13^C-NMR (50 MHz): 168.7 (C=O); 154.0 (C-5); 151.7 (C-2); 115.2 (C-1); 138.2 (C-1′); 131.6 (C-4′); 129.3 (C-2′, C-6′); 128.5 (C-5′, C-3′); 121.2 (C-3); 118.4 (C-4); 111.3 (C-6); 55.8 (OCH_3_); 41.9 (C-7′) [16]. HRMS (MALDI) calculated for C_15_H_14_ClNO_3_ [M + H]^+^: 327.0512; found 327.0516.

*N-(4-Chlorobenzyl)-3-methyl-4-nitrobenzamide* (**20**). Crystalline solid; yield: 41% (143 mg), m.p. 148–151 °C, IR ν_max_ (cm^−1^): 3278 (NH), 3080 (CH sp^2^), 1637 (C=O), 1587 and 1423 (aromatic C=C), 1521 and 1355 (NO_2_ arom), 1089 (stretch C-Cl). ^1^H-NMR (DMSO-*d*_6_): 9.31 (t, *J =* 5.8 Hz, 1H, O=C-NH), 8.06 (dd, *J =* 8.4, 1H; H-5), 7.96 (s, 1H, H-2), 7.89 (dd, *J =* 8.4, 1.7 Hz, 1H; H-6), 7.42–7.31 (m, 4H, H-2′, H-3′, H-5′, H-6′), 4.47 (dd, *J =* 5.9, 1.7 Hz, 2H, H-7′), 2.56–2.51 (m, 3H; CH_3_). ^13^C-NMR (DMSO-*d*_6_): 164.8 (C=O); 150.5 (C-4); 138.3 (C-1); 138.1 (C-1′); 132.9 (C-4′); 131.8 (C-2′, C-6′); 131.5 (C-3); 129.3 (C-3′, C-5′); 128.4 (C-2); 126.2 (C-5); 124.6 (C-6); 42.3 (C-7′); 19.5 (**C**H_3_) [16]. HRMS (MALDI) calculated for C_15_H_13_ClN_2_NaO_3_ [M + Na]^+^: 327.0512; found 327.0516.

*N-(4-Fluorobenzyl)-4-hydroxy-3-methoxybenzamide* (**21**). 4-Fluorbenzylamine was used as the reagent. White amorphous solid; yield: 21% (90 mg), m.p. 161–165 °C, IR ν_max_ (cm^−1^): 3304 (O-H), 3078 (N-H), 1631 (C=O), 1593 and 1423 (aromatic C=C), 1116 (C-F stretch). ^1^H-NMR (CDCl_3_): 9.60 (s, 1H; OH), 8.83 (t, *J =* 5.8 Hz, 1H; NH), 7.52–7.28 (m, 4H; H-2, H-6, H-2′, H-6′), 7.22–7.05 (m, 2H; H-3′, H-5′), 6.81 (d, *J =* 8.2 Hz, 1H; H-5), 4.43 (d, *J =* 5.8 Hz, 2H; H-7′), 3.80 (s, 3H; OMe). ^13^C-NMR (CDCl_3_): 166.0 (C=O), 161.1 (d, *J =* 242.0 Hz; C-4′), 149.6 (C-4), 147.2 (C-3), 136.2 (C-1′), 129.2 (C-2′, C-6′), 125.3 (C-1), 120.9 (C-6), 114.9 (C-3′, C-5′), 115.2 (C-5), 111.3 (C-2), 55.7 (OMe), 42.0 (C-7′) [16]. HRMS (MALDI) calculated for C_15_H_14_FNNaO_3_ [M + Na]^+^: 298.0855; found 298.0882.

*N-(4-Bromobenzyl)-4-hydroxy-3-methoxybenzamide* (**22**). 4-Bromobenzylamine was used as reactant. Red amorphous solid; yield: 63% (252 mg), m.p. 119–122 °C. IV ν_max_ (cm^−1^): 3304 (OH), 3078 (NH), 3003 (CH sp^2^), 1631 (C=O), 1593 and 1423 (aromatic C=C), 1072 (stretching C-Br). ^1^H-NMR (DMSO-*d*_6_): 7.43 (d, *J =* 8.4 Hz, 3H; H-2, H-3′, H-5′), 7.29–7.12 (m, 3H; H-6, H-2′, H-6), 6.88 (d, *J =* 8.2 Hz, 1H; H-5), 6.65 (t, *J =* 5.2 Hz, 1H; NH), 6.21 (s, 1H; OH), 4.54 (d, *J =* 5.8 Hz, 2H, H-7′), 3.88 (s, 3H; OMe). ^13^C-NMR (DMSO-*d*_6_): 167.1 (C=O), 148.9 (C-4), 146.7 (C-3), 137.4 (C-1′), 131.7 (C-3′, C-5′), 129.4 (C-2′, C-6′), 126.1 (C-1), 119.8 (C-6, C-4′), 113.9 (C-5), 110.4 (C-2), 56.0 (OMe), 43.4 (C-7′) [16]. HRMS (MALDI) calculated for C_15_H_14_BrNO_3_ [M]^+^: 335.0157; found 335.0156.

*Procedure 2:* *Preparation of 4-((4-chlorobenzyl) carbamoyl)-2-methoxyphenyl benzoate* (**23**): To a 100 mL flask equipped with magnetic stirring, was added a sodium hydroxide solution 10% (0.51 mL, 0.1275 mmol NaOH) 0.100 g of the amide **15** (0.3400 mmol). Then, benzoyl chloride (0.04 mL, 0.343 mmol) was added in drops. The reaction was subjected to constant agitation for a period of one hour at room temperature. The reaction mixture was then poured into a separation funnel and extraction was completed with dichloromethane (3 × 15 mL), organic phase treated with sodium carbonate (Na_2_CO_3_) 5% (2 × 5 mL), and dried with anhydrous sodium sulfate. After filtration, the solution was concentrated under reduced pressure [18]. The product was purified by silica gel column chromatography using a mixture EtOAc:Hex (65:35) as mobile phase to give a crystalline solid compound, yield: 76% (103 mg), m.p. 122–125 °C, IR ν_max_ (cm^−1^): 3334 (N-H), 3000 (C-H sp^2^), 1734 and 1637 (C=O), 1607 and 1425 (aromatic C=C), 1089 (stretching C-Cl). ^1^H-NMR (CDCl_3_): 9.17 (t, *J =* 5.9 Hz, 1H, NH), 8.13 (d, *J =* 7.1 Hz, 2H, H-2″, H-6″), 7.84–7.20 (m, 10H; H-2, H-5, H-6, H-2, H-3′, H-5′, H-6′, H-3″, H-4″, H-5″), 4.50 (d, *J =* 5.8 Hz, 2H; H-7′). 3.81 (s, *J =* 6.0 Hz, 3H; OMe). ^13^C-NMR (CDCl_3_): 165.9 (O=C-O), 164.2 (O=C-N), 151.2 (C-3), 142.1 (C-4), 139.1 (C-1′), 134.6 (C-2′), 133.6 (C-1), 134.6 (C-6′), 131.1 (C-4′), 130.3 (C-2″, C-4″, C-6″), 129.6 (C-3″, C-5″), 128.8 (C-1″), 128.7 (C-3′, C-5′), 123.4 (C-5), 120.4 (C-6), 112.2 (C-2), 42.5 (C-7′), 56.4 (OMe) [19]. HRMS (MALDI) calculated for C_22_H_18_ClNNaO_4_ [M + Na]^+^: 418.0822; found 418.0809. The following compounds were similarly prepared:

*4-((4-Chlorobenzyl)carbamoyl)-2-methoxyphenyl 2-phenylacetate* (**24**). Phenylacetyl chloride (0.05 mL, 0.343 mmol) was used. The reaction was subjected to constant agitation for a period of two hours at room temperature and finally extracted. Crystalline solid; yield: 78% (110 mg), m.p.: 141–145 °C, IR ν_max_ (cm^−1^): 3331 (N-H), 3050 (C-H sp^2^), 1761 and 1633 (C=O), 1602 and 1456 (aromatic C=C), 1033 (stretching C-Cl). ^1^H-NMR (CDCl_3_): 9.12 (t, *J =* 5.9 Hz, 1H; NH), 7.61 (d, *J=* 1.7 Hz, 1H; H-2), 7.52 (dd, *J =* 8.2, 1.8 Hz, 1H; H-6), 7.43–7.26 (m, 10H; H-2′, H-3′, H-5′, H-6′, H-2″, H-3″, H-4″, H-5″, H-6″), 7.20 (d, *J =* 8.2 Hz, 1H; H-5), 4.47 (d, *J =* 5.9 Hz, 2H; H-7′), 3.98 (s, 2H, CH_2_-C_6_H_5_), 3.80 (s, 3H; OMe). ^13^C-NMR (CDCl_3_): 169.6 (O=C-O), 165.7 (O=C-N), 150.9 (C-3), 142.0 (C-4), 138.9 (C-1′), 134.1 (C-1″), 133.3 (C-4′), 131.6 (C-1), 129.8 (C-2′, C-6′), 128.7 (C-2″, C-6″), 128.6 (C-3″, C-5″), 127.3 (C-4″), 127.3 (C-3′, C-5′), 122.9 (C-5), 120.2 (C-6), 112.0 (C-2), 56.3 (OCH_3_), 42.4 (C-7′), 40.09 (**C**H_2_-C_6_H_5_) [20]. HRMS (MALDI) calculated for C_23_H_20_ClNNaO_4_ [M + Na]^+^: 432.0979; found 432.0914.

*4-((4-Chlorobenzyl)carbamoyl)-2-methoxyphenyl valerate* (**25**). Valeryl chloride (0.05 mL, 0.343 mmol) was used. The reaction was subjected to constant agitation for a period of five hours at room temperature and finally extracted. White amorphous solid; yield: 43% (55 mg), m.p. 97–100 °C, IR ν_max_ (cm^−1^): 3253 (N-H), 3088 (C-H sp^2^), 1761 and 1631 (C=O), 1602 and 1450 (aromatic C=C), 1031 (stretching C-Cl). ^1^H-NMR (CDCl_3_): 7.44 (s, 1H; H-2); 7.31–7.15 (m, 5H; H-6, H-2′, H-3′, H-5′, H-6′), 6.96 (d, *J =* 8.2 Hz, 1H; H-5), 6.79 (t, *J =* 5.5 Hz, 1H; NH), 4.49 (d, *J =* 5.8 Hz, 2H; H-7′), 3.78 (s, 3H; OCH_3_), 2.56 (t, *J =* 7.4 Hz, 2H; H-1″), 1.71 (quint, *J =* 7.7 Hz, 2H; H-2″), 1.42 (sex, *J =* 7.3 Hz, 2H; H-3″), 0.94 (t, *J =* 7.2 Hz, 3H; Me). ^13^C-NMR (CDCl_3_): 171.7 (O=C-O), 166.6 (O=C-N), 151.3 (C-3), 142.4 (C-4), 136.7 (C-1′), 133.2 (C-1), 132.8 (C-4′), 129.1 (C-2′, C-6′), 128.7 (C-3′, C-5′), 122.6 (C-5), 118.7 (C-6), 111.8 (C-2), 55.9 (OMe), 43.3 (C-7′), 33.7 (C-1″), 26.9 (C-2″), 22.1 (C-3″), 13.7 (Me) [20]. HRMS (MALDI) calculated for C_20_H_22_ClNNaO_4_ [M + Na]^+^: 398.1135; found 398.1118.

*4-((4-Chlorobenzyl) carbamoyl)-2-methoxyphenyl 3-bromobenzoate* (**26**). 3-Bromobenzoyl chloride (0.05 mL, 0.343 mmol) was employed. The reaction was subjected to constant agitation for a period of one hour at room temperature and finally extracted. White amorphous solid; yield: 86% (140 mg), m.p. 143–145 °C. IV νmax (cm^−1^): 3334 (N-H), 3050 (C-H sp^2^), 1734 and 1637 (C=O), 1602 and 1490 (aromatic C=C), 1089 (stretching C-Cl). ^1^H-NMR (CDCl_3_): 8.30 (t, *J =* 1.7 Hz, 1H; H-2″), 8.14–8.03 (m, 1H; H-4″), 7.81–7.71 (m, 1H; H-6″), 7.51 (d, *J =* 1.8 Hz, 1H; H-2), 7.44–7.15 (m, 6H; H-6, H-2′, H-3′, H-5′, H-60′, H-5″), 7.09 (d, *J =* 8.2 Hz, 1H; H-5), 6.81 (t, *J =* 5.7 Hz, 1H; NH), 4.53 (d, *J =* 5.8 Hz, 2H; H-7′), 3.80 (s, 3H; OMe). ^13^C-NMR (CDCl_3_): 166.6 (O=C-O), 163.2 (O=C-N), 151.4 (C-3), 142.2 (C-4), 136.7 (C-4″), 136.6 (C-1′), 133.3 (C-1), 133.2 (C-1″), 133.2 (C-2″), 130.7 (C-4′), 130.1 (C-5″), 129.1 (C-2′, C-6′), 128.8 (C-3′, C-5′, C-6′′), 122.7 (C-3″), 122.6 (C-5), 118.7 (C-6), 112.0 (C-2), 43.4 (C-7′), 56.0 (OCH_3_) [21]. HRMS (MALDI) calculated for C_20_H_22_ClNO_4_ [M]^+^: 495.9927; found 495.9912.

*Procedure 3: Preparation of 4-((4-chlorobenzyl) carbamoyl)-2-methoxyphenyl acetate* (**27**). For the acetylation of **15**, to a 50 mL flask, equipped with magnetic stirrer was added the chlorinated vanillic amide **15** (0.1000 g, 0.034 mmol), pyridine (0.13 mL, 0.15924 mmol) and acetic anhydride (0.08 mL, 0.8111 mmol). The reaction mixture was subjected to constant magnetic stirring for 24 h. The first step of the reaction product extraction was then carried out by pouring into ice water (30 mL) in a separation funnel using ethyl acetate as extractor solvent (3 × 10 mL). The organic phase was treated with saturated copper sulfate solution (3 × 20 mL). The ethyl acetate phase was washed with water (3 × 30 mL) and dried with anhydrous sodium sulfate (Na_2_SO_4_). Subsequently, the organic phase was filtered and concentrated by rotary evaporation [22]. The product was purified by silica gel column chromatography using EtOAc:Hex (65:35) as the mobile phase system to give a crystalline solid; yield: 98% (159 mg), m.p. 117–119 °C, IR ν_max_ (cm^−1^): 3446 (N-H), 3088 (C-H sp^2^), 1770 and 1635 (C=O), 1602 and 1421 (aromatic C=C), 1089 (stretching C-Cl). ^1^H-NMR (CDCl_3_): 7.43 (d, *J =* 1.9 Hz, 1H; H-2), 7.33–7.12 (m, 4H; H-2, H-3′, H-5′, H-6′), 7.03–6.86 (m, 1H; H-6), 6.88–6.69 (m, 1H; H-5) ,4.47 (d, *J =* 5.8 Hz, 2H, H-7′), 3.77 (s, 3H; Me), 2.27 (s, 3H; Me). ^13^C-NMR (CDCl_3_): 168.8 (O=C-O), 166.6 (O=C-N), 151.3 (C-3), 142.3 (C-4), 136.6 (C-1′), 133.2 (C-1), 132.9 (C-4′), 129.0 (C-2′, C-6′), 128.7 (C-3′, C-5′), 122.6 (C-5), 118.7 (C-6), 111.8 (C-2), 55.9 (OMe), 43.3 (C-7′), 20.6 (Me) [19]. HRMS (MALDI) calculated for C_17_H_16_ClNNaO_4_ [M + Na]^+^: 356.0666; found 356.0664.

*Procedure 4:* *Preparation of N-(4-chlorobenzyl)-3-methoxy-4-(4-methylphenetoxy)benzamide* (**28**). In a 100 mL flask equipped with magnetic stirring vanillic amide **15** (0.1000 g, 0.3400 mmol) was added to a solution of acetone (4 mL) together with K_2_CO_3_ (0.1388 g, 1.0046 mmol) and 4-methylbenzyl bromide (0.08 mL, 0.6011 mmol) under reflux (60 °C) for 16 h. After the reaction, the solvent was removed under reduced pressure. A solution of CH_2_Cl_2_:H_2_O was poured onto the product, placed in a separation funnel and extracted with three portions of CH_2_Cl_2_ (10 mL each). The organic phase was washed 1 N NaOH (3 × 10 mL) and dried with Na_2_SO_4_, filtered and concentrated by rotary evaporation. The product was purified by silica gel column chromatography using EtOAc:Hex (65:35) as the mobile phase system to give a white amorphous solid; yield: 30% (42 mg), m.p. 126–129 °C, IR ν_max_ (cm^−1^): 3290 (N-H), 3001 (C-H sp^2^), 1629 (C=O), 1600 and 1490 (aromatic C=C), 1029 (stretching C-Cl). ^1^H-NMR (CDCl_3_): 7.44 (s, 1H; H-2), 7.32–7.07 (m, 9H; H-6, H-2′, H-3′, H-5′, H-6′, H-2″, H-3″, H-5″, H-6″), 6.80 (d, *J =* 8.4 Hz, 1H; H-5), 6.49 (t, *J =* 5.2 Hz, 1H, NH), 4.56 (d, *J =* 5.8 Hz, 2H; H-7′), 4.18 (t, *J =* 7.6 Hz, 2H; CH_2_-O), 3.88 (s, 3H; OCH_3_), 3.11 (t, *J =* 7.6 Hz, 2H; C**H_2_**-C_6_H_5_). ^13^C-NMR (CDCl_3_): 167.0 (O=C-N), 151.2 (C-3), 149.3 (C-4), 136.9 (C-1′), 136.2 (C-4″), 134.3 (C-1″), 133.3 (C-4′), 129.2 (C-2′, C-6′), 129.1 (C-3″, C-5″), 128.9 (C-3′, C-5′), 128.8 (C-2″, C-6″), 126.7 (C-1), 119.3 (C-6), 111.6 (C-2), 111.1 (C-5), 69.9 (CH_2_-O), 56.1 (OMe), 43.3 (C-7′), 35.1 (CH_2_-C_6_H_5_), 21.0 (Me) [23]. HRMS (MALDI) calculated for C_24_H_24_ClNNaO_3_ [M + Na]^+^: 432.1342; found 432.1328.

*Procedure 5: Preparation of N-(4-chlorobenzyl)-4-(4-hydroxyphenoxy)-3-methoxybenzamide* (**29**). In a 100 mL flask equipped with magnetic stirring, vanillic amide **15** (0.1000 g, 0.3400 mmol) was added to a solution of DMF (3.43 mL), K_2_CO_3_ (0.0711 g, 0.5142 mmol), and 4-hydroxyphenyl bromide (0.0827 g, 0.4113 mmol), and left stirring for 24 h at room temperature. After the reaction, the product was extracted in a separation funnel with CH_2_Cl_2_ (3 × 10 mL). The extraction solution was washed with distilled water (3 × 10 mL), and then with 10% NaOH (10 mL). The solution was treated with Na_2_SO_4_, filtered, and concentrated by rotary evaporation [24]. The product was purified by silica gel column chromatography using EtOAc:Hex (65:35) as the mobile phase system to afford a colorless oil; yield: 75% (106 mg), IR ν_max_ (cm^−1^): 3350 (OH) 3016 (CH sp^2^), 1645 (C=O), 1600 and 1489 (aromatic C=C), 1014 (stretching C-Cl). ^1^H-NMR (CDCl_3_): 7.95 (s, 1H; NH), 7.44 (s, 1H; H-2), 7.30–6.94 (m, 8H; H-5, H-6, H-2′, H-3′, H-5′, H-6′, H-2″, H-6″), 6.79 (dd, *J =* 8.5, 2.6 Hz, 2H; H-3″, H-5″), 4.54 (d, *J =* 5.8 Hz, 2H; H-7′), 4.14 (t, *J =* 7.6 Hz, 2H; CH_2_-O), 3.82 (s, 3H; OMe), 3.04 (t, *J =* 7.5 Hz, 2H; C**H**_2_-C_6_H_5_). ^13^C-NMR (CDCl_3_): 167.5 (O=C-N), 155.0 (C-4″), 151.3 (C-4), 149.2 (C-3), 136.7 (C-1′), 133.3 (C-4′), 130.0 (C-2′, C-6′), 129.1 (C-2″, C-6″), 128.8 (C-3′, C-5′), 128.9 (C-1″), 126.4 (C-1), 119.5 (C-6), 115.6 (C-3″, C-5″), 111.6 (C-2), 111.2 (C-5), 70.1 (CH_2_-O), 56.1 (OCH_3_), 43.4 (C-7′), 34.8 (**C**H_2_-C_6_H_5_) [23]. HRMS (MALDI) calculated for C_23_H_22_ClNNaO_4_ [M + Na]^+^: 434.1135; found 434.1123.

*Preparation of N-(4-chlorobenzyl)-3-methoxy-4-propoxybenzamide* (**30**). This product was prepared according to procedure 4, using 1-propyl bromide (0.05 mL, 0.4102 mmol) under reflux (60 °C), for 16 h and finally extracted to give after the silica gel column chromatography a white amorphous solid; yield: 54% (62 mg), m.p. 124–126 °C. ν_max_ IR (cm^−1^): 3296 (N-H), 1631 (C=O), 1581 to 1450 (aromatic C=C), 1014 (C-Cl stretch). ^1^H-NMR (CDCl_3_): 7.40 (d, *J =* 1.8 Hz, 1H; H-2), 7.31–7.22 (m, 5H; H-6, H-2′, H-3′, H-5′, H-6′), 6.81 (d, *J =* 8.4 Hz, 1H; H-5), 6.74 (bs, 1H; NH), 4.55 (d, *J =* 5.8 Hz, 2H; H-7′), 3.98 (t, *J =* 6.8 Hz, 2H; H-1″), 3.86 (s, 3H; OMe), 2.04–1.76 (m, 2H; H-2″), 1.03 (t, *J =* 7.4 Hz, 3H; H-3″). ^13^C-NMR (CDCl_3_): 167.0 (O=**C**-N), 151.4 (C-4), 149.2 (C-3), 137.0 (C-1′), 133.1 (C-4′), 129.1 (C-2′, C-6′), 128.7 (C-3′, C-5′), 126.3 (C-1), 119.4 (C-6), 110.9 (C-2), 111.4 (C-5), 70.4 (C-1″), 56.0 (OCH_3_), 43.3 (C-7′), 22.3 (C-2″), 10.3 (C-3″) [25]. HRMS (MALDI) calculated for C_18_H_20_ClNNaO_3_ [M + Na]^+^: 356.1029; found 356.1038.

*Preparation of N-(4-chlorobenzyl)-4-isopropoxy-3-methoxybenzamide* (**31**). This product was prepared according to procedure 5, using 2-propyl bromide (0.05 mL, 0.4102 mmol), at room temperature and left stirring for 24 h and finally extracted to give, after silica gel column chromatography purification using EtOAc:Hex as the gradient mobile phase system, increasing in polarity and an isocratic EtOAc:Hex (65:35) system after elution of the purified product a white amorphous solid; yield: 54% (62 mg), m.p. 142–147 °C. IV ν_max_ (cm^−1^): 3284 (N-H), 3078 (C-H sp^2^), 1631 (C=O), 1598 and 1455 (aromatic C=C), 1031 (stretching C-Cl). ^1^H-NMR (CDCl_3_): 7.40 (d, *J =* 1.8 Hz, 1H; H-6), 7.26–7.18 (m, 5H; H-2, H-2′, H-3′, H-5′, H-6′), 6.79 (d, *J =* 8.4 Hz, 2H; H-5), 6.79 (bs, *J =* 8.4 Hz, 2H; NH), 4.51 (sept, *J =* 6.2 Hz, 1H; H-2″), 4.51 (d, *J =* 5.7 Hz, 2H; H-7′), 3.81 (s, 3H; OMe), 1.33 (d, *J =* 6.0 Hz, 6H; H-1″; H-3″). ^13^C-NMR (CDCl_3_): 167.1 (O=C-N), 150.3 (C-4), 150.0 (C-3), 137.0 (C-1′), 133.2 (C-4′), 129.1 (C-2′, C-6′), 128.7 (C-3′, C-5′), 126.5 (C-1), 119.4 (C-6), 113.6 (C-5), 111.2 (C-2), 71.2 (C-1″), 56.0 (OCH_3_), 43.3 (C-7′), 21.9 (Me) [25]. HRMS (MALDI) calculated for C_18_H_20_ClNNaO_3_ [M + Na]^+^: 356.1059; found 356.1027.

*Preparation of N-(4-chlorobenzyl)-4-(decyloxy)-3-methoxybenzamide* (**32**). The product was prepared according to procedure 4, using 1-decyl bromide (0.05 mL, 0.4102 mmol) under reflux (60 °C) for 16 h and finally extracted to give after silica gel column chromatography a white amorphous solid; yield: 81% (120 mg), m.p. 110–105 °C, IR ν_max_ (cm^−1^): 3305 (N-H), 3000 (C-H sp^2^), 1627 (C=O), 1602 and 1508 (aromatic C=C), 1035 (stretching C-Cl). ^1^H-NMR (CDCl_3_): 7.42 (s, 1H; H-6), 7.26 (s, 5H; H-2, H-2′, H-3′, H-5′, H-6′), 6.54 (s, 1H; H-5), 4.56 (d, *J =* 5.8 Hz, 2H; H-7′), 4.01 (t, *J =* 6.8 Hz, 2H; CH_2_-C_6_H_5_), 3.86 (s, 3H; OMe), 1.83 (dt, *J =* 14.1, 6.9 Hz, 2H; CH_2_-O), 1.74–1.15 (m, 14H; (CH_2_)_7_), 0.86 (t, *J =* 6.1 Hz, 3H; CH_3_). ^13^C-NMR (CDCl_3_): 167.2 (O=C-N), 151.7 (C-4), 149.4 (C-3), 137.1 (C-1′), 133.4 (C-4′), 129.3 (C-2′, C-6′), 128.9 (C-3′, C-5′), 126.5 (C-1), 119.5 (C-6), 111.6 (C-2), 111.1 (C-5), 69.2 (CH_2_O), 56.2 (OMe), 43.5 (C-7′), 32.0 (**C**H_2_CH_2_O), 29.7–22.8 ((**C**H_2_)_7_CH_2_O), 14.3 (**C**H_3_). [26]. HRMS (MALDI) calculated for C_18_H_20_ClNaNO_3_ [M + Na]^+^: 454.2125; found 454.2119.

### 3.3. Antifungal Activity

#### 3.3.1. Microbiological Strains

The microorganisms used in microbiological tests were strains of *Candida albicans* (ATCC 76645, LM-106 and LM-23), *C. krusei* (LM-13 and LM-656) and *C. tropicalis* (ATCC-13803 and ML-36). The strains were respectively acquired from the Adolfo Lutz Institute in São Paulo (Brazil), and from the Federal University Pharmaceutical Science Mycology Laboratories of São Paulo and Paraiba. The yeast strains were maintained in appropriate medium of Sabouraud Dextrose Broth-SDB prepared and used according to manufacturer’s instructions (Difco Laboratories, MA, USA), and stored at 4 °C and 35 °C. The microorganism suspension was prepared according to McFarland tube 0.5, and adjusted by means of a spectrophotometer (Leitz-Photometer 340-800, Ernst Leitz, Wetzlar, Germany) at 90% T (530 nm) corresponding to approximately 10^6^ CFU mL^−1^ [29,30,33].

#### 3.3.2. Determination of the Minimum Inhibitory Concentration (MIC)

The MIC value was determined by microdilution method using 96 well “U” shaped micro-titer plates in duplicate. In each well of the plate 100 µL of twice concentrated SDB liquid medium was added. Then, 100 mL of product solution (also doubly concentrated) was placed in the first row of plate wells. Through serial dilution (ratio of two), the concentrations of 1024 µg/mL to 64 µg/mL were obtained, such that in the first line of the plate was the highest concentration and in the latter, the lower concentrations. Finally, 10 µL of inoculum was added to the wells in each plate column that specifically referred to a strain. The same was also done in the culture medium with the fungal drug nystatin (100 IU). The plates were incubated at 37 °C for 24–48 h. For each strain, the MIC was defined as the lowest concentration capable of inhibiting fungal growth in the wells as visually observed compared with the control. All tests were performed in duplicate and the results were expressed as a geometric mean of the MIC values obtained in both tests [34].

### 3.4. Qualitative-Structure Activity Relationship of Antimicrobial Activity in Amides 

#### 3.4.1. Volsurf Descriptors

The molecular structures in three dimensions (3D) were used as input data to the Volsurf + v program. 1.0.7, and subjected to molecular interaction fields (MIC) to generate descriptors using the following probes: N1 (amide hydrogen-nitrogen bond donor probe), O (hydrogen-oxygen carbonyl bond acceptor probe), OH2 (probe water), and DRY (hydrophobic probe). Descriptors not using probes were generated to create a total of 128 descriptors [35].

#### 3.4.2. Models for the Study of Qualitative-Structure Activity Relationship

KNIME 3.1.0 software (3.1.0 KNIME from Konstanz Information Miner, copyright 2003–2014, www.knime.org) [31] was used to perform all of the analyses. For internal validation, cross validation was employed using the “leave-one-out” method. Descriptors were selected, and a model was generated using the training and cross-validation set, using the WEKA nodes [35]. The internal performances of the selected models were analyzed for sensitivity (true positive rate), specificity (true negative rate), and accuracy (overall predictability).

## 4. Conclusions

A number of *N*-(4-halobenzyl)amides **1**–**32** were prepared and their antifungal potential was evaluated in screening experiments carried out with seven fungal strains. Compounds **2**, **3**, **5**, **6**, **10**, **14**, and **15** (MICs = 256 and 512 μg·mL^−1^) showed considerable antifungal activity against all tested strains of the genus *Candida*. According to the SAR, it was observed that disubstituted amides, such as *meta*- and *para*-hydroxylated or *meta*-methoxylated/*para*-hydroxylated or 3,4,5-trihydroxylated *N*-(4-halobenzyl)amides have better antifungal activity against *Candida* strains. In addition, the presence of the nitro group in an *ortho* position in the benzene ring contributes to the antifungal activity. This study with haloamides could help in the development of future therapeutic approaches to the growing problem of microbial pathogens via the discovery of novel antifungal agents.

## Figures and Tables

**Table 1:** Data for amides derived from cinnamic acid and benzoic acid.

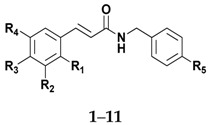					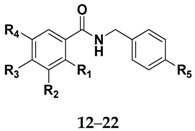			
Compound	R_1_	R_2_	R_3_	R_4_	R_5_	Molecular Formula	Reaction Time (h)	Yield (%)
**1**	-	-	-	-	Cl	C_16_H_14_ClNO	3	75
**2**	-	OH	OH	-	Cl	C_16_H_14_ClNO_3_	7	70
**3**	-	OMe	OH	-	Cl	C_17_H_16_ClNO_3_	4	81
**4**	-	-	OMe	-	Cl	C_17_H_16_ClNO_2_	2	91
**5**	OH	-	-	-	Cl	C_16_H_14_ClNO_2_	5	79
**6**	-	OH	-	-	Cl	C_16_H_14_ClNO_2_	4	76
**7**	-	-	OH	-	Cl	C_16_H_14_ClNO_2_	3	63
**8**	-	-	Cl	-	Cl	C_16_H_13_Cl_2_NO	2	71
**9**	-	OMe	OH	OMe	Cl	C_18_H_18_ClNO_4_	6	60
**10**	NO_2_	-	-	-	Cl	C_16_H_13_ClN_2_O_3_	2	79
**11**	-	OMe	OMe	OMe	Cl	C_19_H_20_ClNO_4_	3	86
**12**	-	-	-	-	Cl	C_14_H_12_ClNO	3	65
**13**	-	-	C_6_H_5_		Cl	C_20_H_16_ClNO	6	56
**14**	-	OH	OH	OH	Cl	C_14_H_12_ClNO_4_	3	21
**15**	-	OMe	OH	-	Cl	C_15_H_14_ClNO_3_	6	44
**16**	-	OMe	OH	OMe	Cl	C_16_H_16_ClNO_4_	3	50
**17**	-	-	OH	-	Cl	C_14_H_12_ClNO_2_	3	73
**18**	-	C(CH_3_)_3_	OH	C(CH_3_)_3_	Cl	C_22_H_28_ClNO_2_	3	54
**19**	-	OH	-	OMe	Cl	C_15_H_14_ClNO_3_	6	60
**20**	-	Me	-	NO_2_	Cl	C_15_H_13_ClN_2_O_3_	4	41
**21**	-	OMe	OH	-	F	C_15_H_14_FNO_3_	2	27
**22**	-	OMe	OH	-	Br	C_15_H_14_BrNO_3_	2	63

**Table 2:** Data from amide **15** derived ethers and esters.

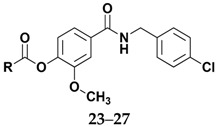		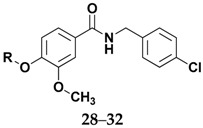		
Compound	R	Molecular Formula	Reaction Time (h)	Yield (%)
**23**	C_6_H_5_-	C_22_H_18_ClNO_4_	1	76
**24**	C_6_H_5_CH_2_-	C_23_H_20_ClNO_4_	2	78
**25**	CH_3_(CH_2_)_3_-	C_20_H_22_ClNO_4_	5	43
**26**	3-Br-C_6_H_4_-	C_22_H_17_BrClNO_4_	1	86
**27**	CH_3_	C_17_H_16_ClNO_4_	24	98
**28**	4-Me-C_6_H_4_CH_2_-	C_24_H_24_ClNO_3_	12	30
**29**	4-OH-C_6_H_4_CH_2_-	C_23_H_22_ClNO_4_	24	75
**30**	CH_3_(CH_2_)_2_-	C_18_H_20_ClNO_3_	12	54
**31**	(CH_3_)_2_CH-	C_18_H_20_ClNO_3_	24	54
**32**	CH_3_(CH_2_)_8_CH_2_-	C_20_H_24_ClNO_3_	12	81

**Table 3:** Evaluation of MIC (µg/mL) of the amides derived from cinnamic acid and benzoic acid in the microdilution broth assay.

MIC ^b^ (µg·mL^−1^)/Yeast							
Compound	*C. albicans* ATCC-76645	*C. albicans* LM-106	*C. albicans* LM-23	*C. tropicalis* ATCC-13803	*C. tropicalis* LM-36	*C. krusei* LM-13	*C. krusei* LM-656
**1**	1024	1024	1024	512	512	+	1024
**2**	256	256	256	512	1024	512	512
**3**	256	256	256	512	512	512	512
**4**	+	+	+	+	+	+	+
**5**	256	256	256	512	512	1024	1024
**6**	256	256	256	512	512	1024	1024
**7**	1024	1024	512	+	+	+	1024
**8**	+	+	+	+	+	+	+
**9**	+	+	+	+	+	+	+
**10**	256	256	256	256	256	512	512
**11**	+	+	+	+	+	+	+
**12**	+	+	+	+	+	+	+
**13**	+	+	+	+	+	+	+
**14**	256	256	512	512	512	256	256
**15**	512	256	256	256	256	256	256
**16**	+	+	+	+	+	+	+
**17**	+	+	+	+	1024	+	256
**18**	+	+	+	+	+	+	+
**19**	+	+	+	+	+	+	+
**20**	+	+	+	+	+	+	+
**21**	512	512	512	+	+	1024	1024
**22**	+	+	+	+	+	+	+
**23**	+	+	+	+	+	+	+
**24**	+	+	+	+	+	+	+
**25**	+	+	+	+	+	+	+
**26**	+	+	+	+	+	+	+
**27**	+	+	+	+	+	+	+
**28**	+	+	+	+	+	+	+
**29**	+	+	+	+	+	+	+
**30**	+	+	+	+	+	+	+
**31**	+	+	+	+	+	+	+
**32**	+	+	+	+	+	+	+
**Nystatin**	3.125	3.125	3.125	3.125	3.125	3.125	3.125

+ Growth of the microorganism ^b^ MIC defined as the lowest concentration that produced 50% reduction in fungal cell growth after 24 h of incubation.

**Table 4:** Summary of training, internal cross-validation, test results and corresponding match results which were obtained using a *leave-one out* validation method in KNIME of the total set of 32 haloamides subjected to antifungal tests.

	*C. albicans* LM-23					*C. tropicalis* ATCC-13803				
	TRAINING			VALIDATION		TRAINING			VALIDATION	
	Samples	Match	%Match	Match	%Match	Samples	Match	%Match	Match	%Match
Active	10	3	30.0	3	30.0	8	2	25.0	2	25.0
Inactive	22	19	86.4	19	86.4	24	20	83.3	20	83.3
Total	32	22	68.8	22	68.8	32	22	68.8	22	68.8
	***C. albicans* ATCC-76645**					***C. tropicalis* LM-36**				
	**TRAINING**			**VALIDATION**		**TRAINING**			**VALIDATION**	
	**Samples**	**Match**	**%Match**	**Match**	**%Match**	**Samples**	**Match**	**%Match**	**Match**	**%Match**
Active	10	3	30.0	3	30.0	9	2	22.2	2	22.2
Inactive	22	19	86.4	19	86.4	23	20	87.0	20	87.0
Total	32	22	68.8	22	68.8	32	22	68.8	22	68.8
	***C. albicans* LM-106**					***C. krusei* LM-656**				
	**TRAINING**			**VALIDATION**		**TRAINING**			**VALIDATION**	
	**Samples**	**Match**	**%Match**	**Match**	**%Match**	**Samples**	**Match**	**%Match**	**Match**	**%Match**
Active	10	3	30.0	3	30.0	11	3	27.3%	3	27.3
Inactive	22	19	86.4	19	86.4	21	19	90.5%	19	90.5
Total	32	22	68.8	22	68.8	32	22	68.8%	22	68.8
	***C. krusei* LM-13**									
	**TRAINING**			**VALIDATION**						
	**Samples**	**Match**	**%Match**	**Match**	**%Match**					
Active	8	3	37.5	3	37.5					
Inactive	24	19	79.2	19	79.2					
Total	32	22	68.8	22	68.8					

## Supplementary Materials

Supplementary materials can be accessed at: http://www.mdpi.com/1420-3049/21/12/1716/s1.
