# Supplementary material for: Synthesis, Antifungal Evaluation and In Silico Study of N-(4-Halobenzyl)amides
Source: Molecules. 2016 Dec 13;21(12):1716. doi: 10.3390/molecules21121716 (PMC6273175; doi:10.3390/molecules21121716)
Supplement: Supplementary file 1 [file molecules-21-01716-s001.pdf]

# Supplementary Material: Synthesis, Antifungal Evaluation and *In Silico* Study of *N*-(4-Halobenzyl)amides

**Ricardo Carneiro Montes, Ana Luiza A. L.Perez, Cássio Ilan S. Medeiros, Marianna Oliveira de Araújo, Edeltrudes de Oliveira Lima, Marcus Tullius Scotti and Damião Pergentino de Sousa**

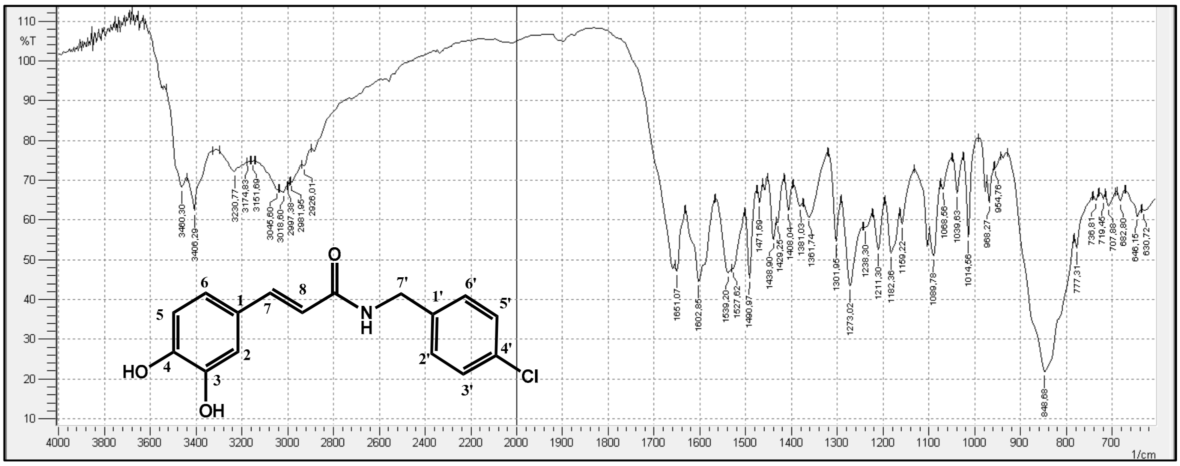


**Figure S1.** Infrared spectrum (KBr, cm$^{-1}$) of (*E*)-*N*-(4-chlorobenzyl)-3-(3,4-dihydroxyphenyl)acrylamide (**2**).

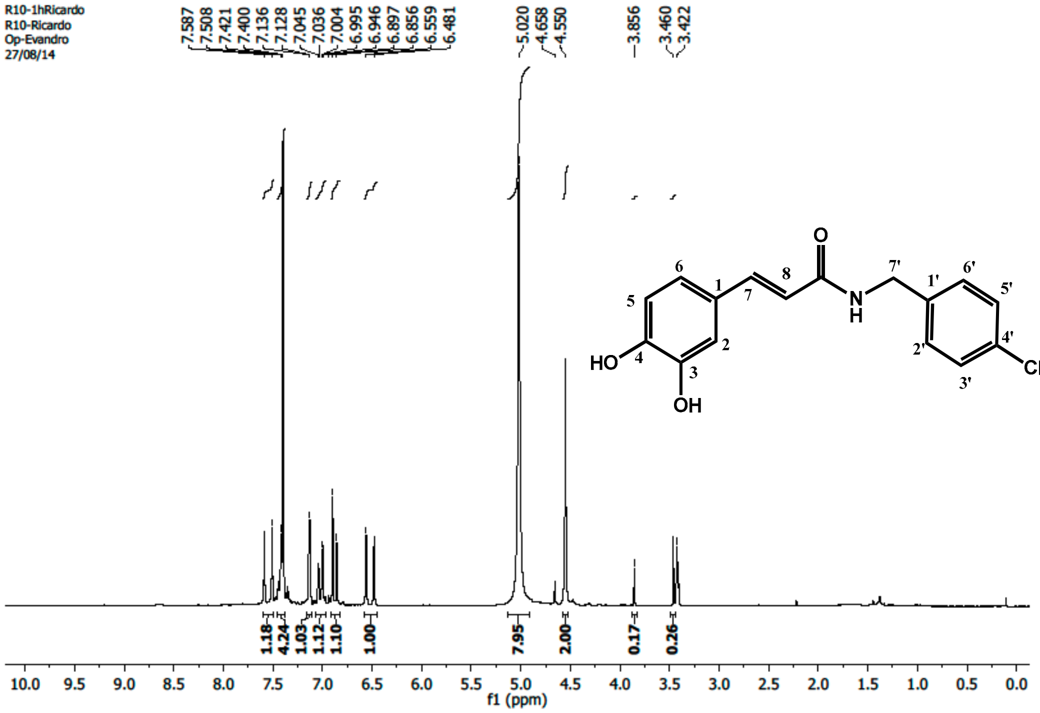


**Figure S2.** $^1$H-NMR spectrum of (*E*)-*N*-(4-chlorobenzyl)-3-(3,4-dihydroxyphenyl)acrylamide (MeOD, 200 MHz).

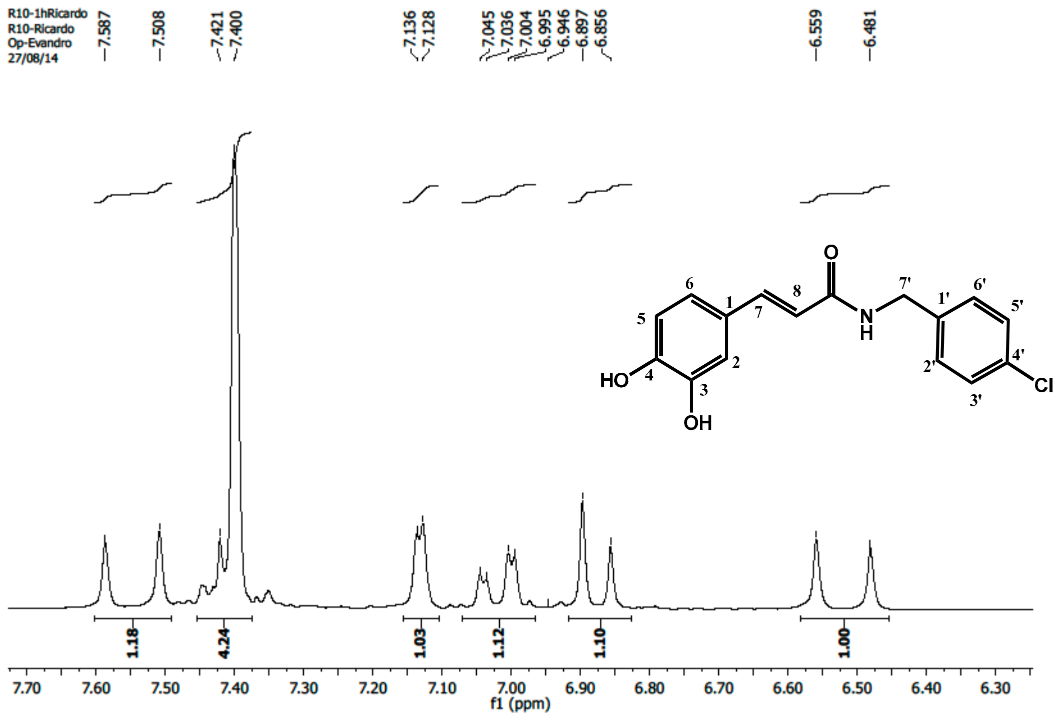


**Figure S3.** Expansion of the $^{1}$H-NMR spectrum of (*E*)-*N*-(4-chlorobenzyl)-3-(3,4-dihydroxyphenyl) acrylamide (**2**) (MeOD, 200 MHz).

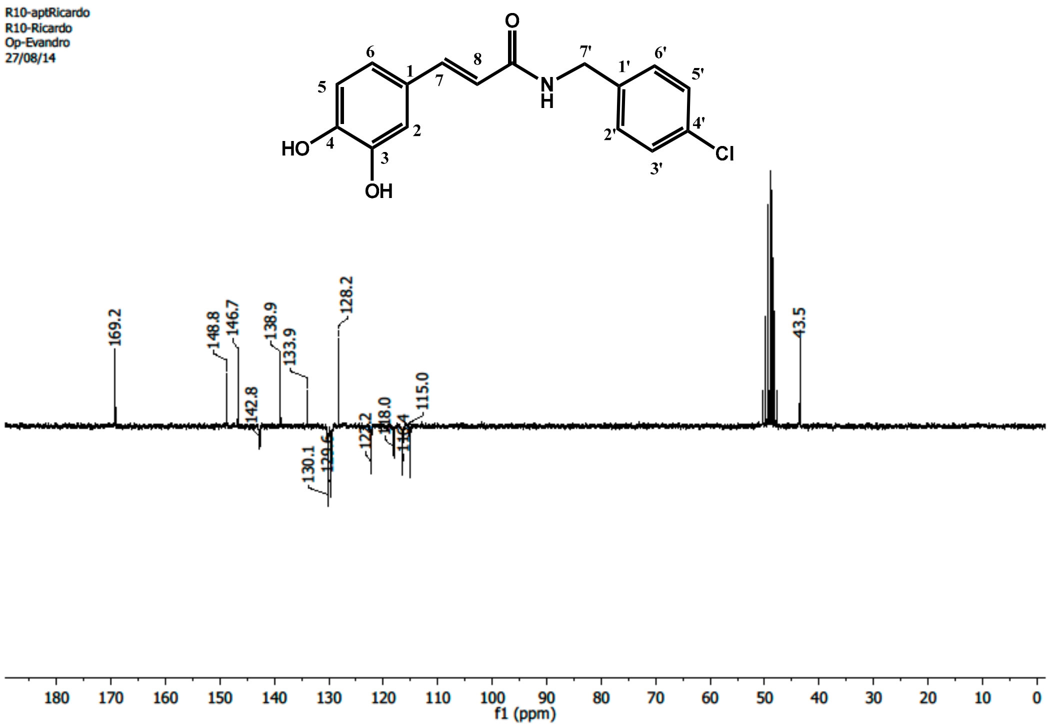


**Figure S4.** $^{13}$C-APT NMR spectrum of (*E*)-*N*-(4-chlorobenzyl)-3-(3,4-dihydroxyphenyl)acrylamide (**2**) (MeOD, 50 MHz).

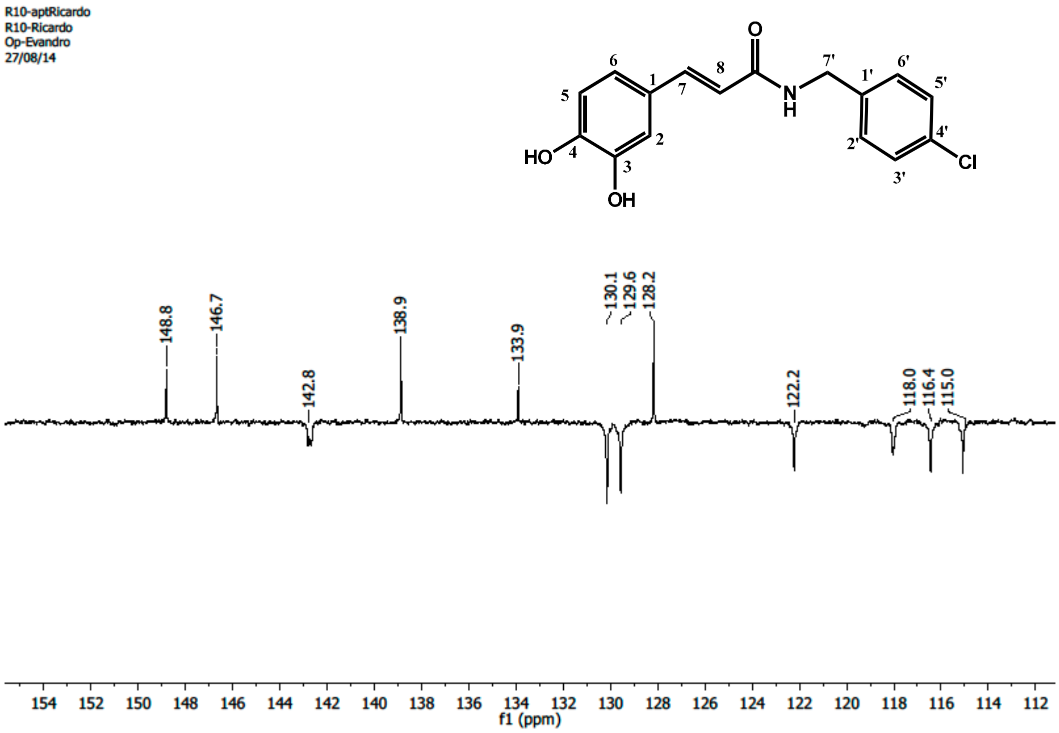


**Figure S5.** Expansion of the $^{13}$C-APT NMR spectrum of (*E*)-*N*-(4-chlorobenzyl)-3-(3,4-dihydroxyphenyl) acrylamide (**2**) (MeOD, 50 MHz).

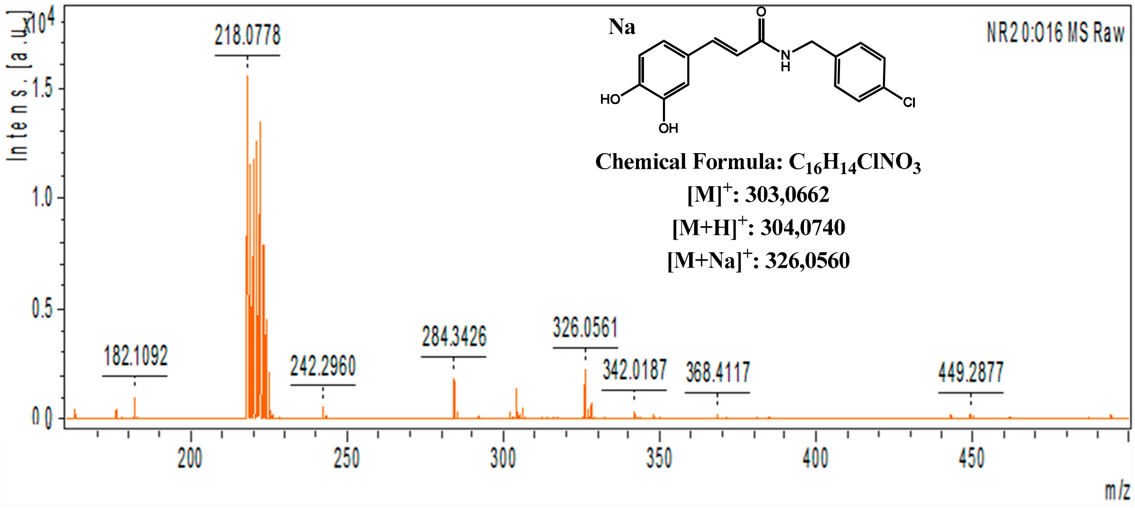


**Figure S6.** High resolution mass spectrum—MALDI of (*E*)-*N*-(4-chlorobenzyl)-3-(3,4-dihydroxyphenyl) acrylamide (**2**).

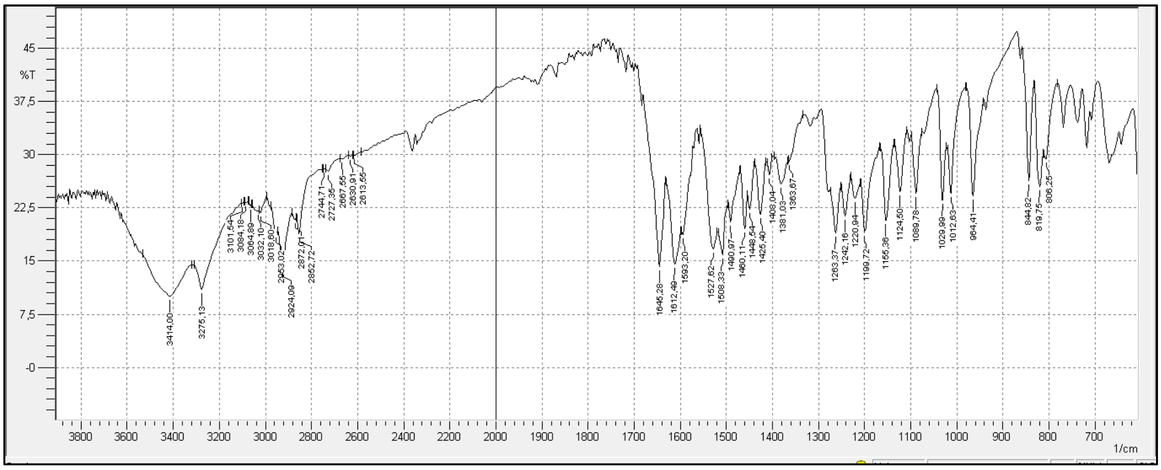


**Figure S7.** Infrared spectrum (KBr, cm$^{-1}$) of (*E*)-*N*-(4-chlorobenzyl)-3-(4-hydroxy-3-methoxyphenyl) acrylamide (**3**).

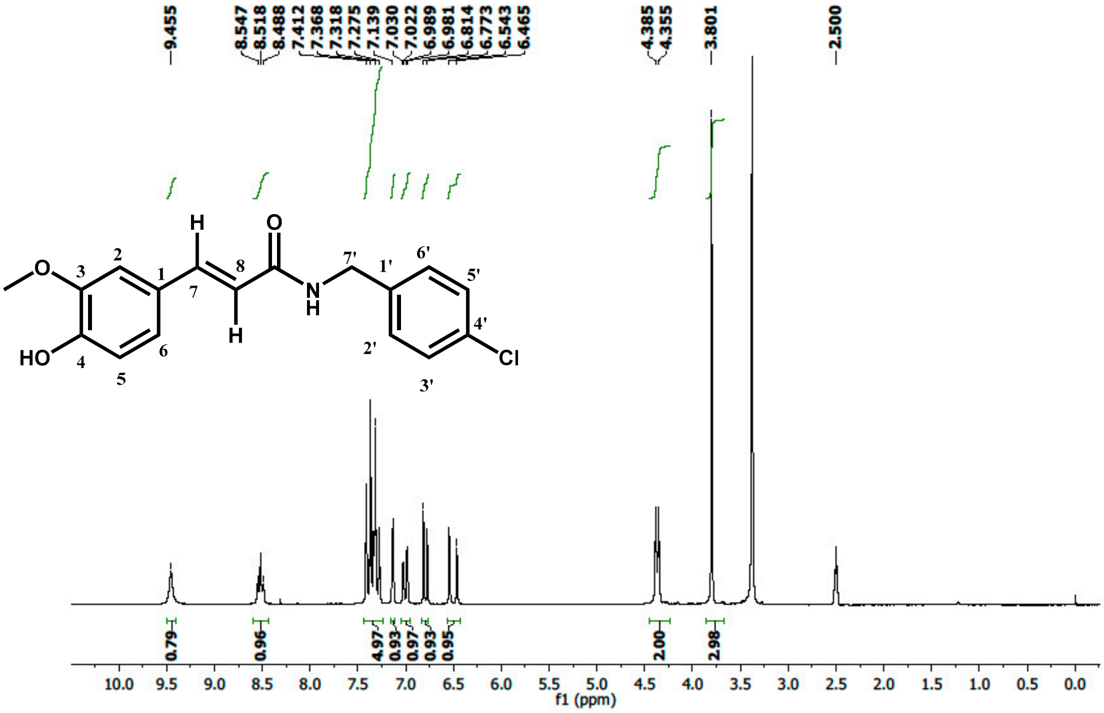


**Figure S8.** $^{1}$H-NMR spectrum of (*E*)-*N*-(4-chlorobenzyl)-3-(4-hydroxy-3-methoxyphenyl)acrylamide (**3**) (DMSO-$d_6$, 200 MHz).

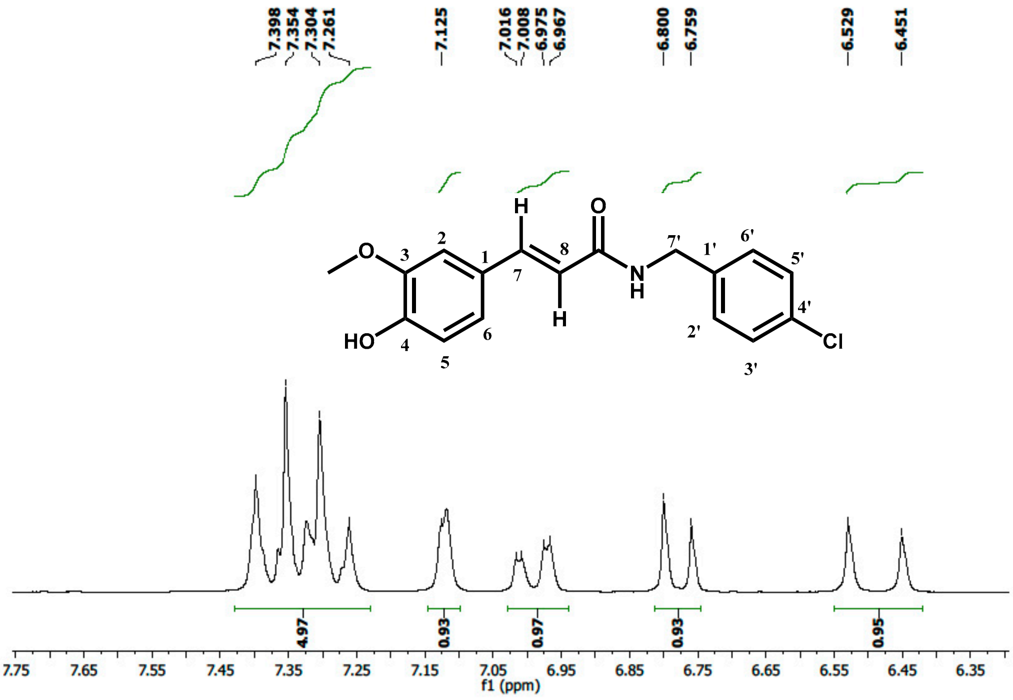


**Figure S9.** Expansion of the $^{1}$H-NMR spectrum of (*E*)-*N*-(4-chlorobenzyl)-3-(4-hydroxy-3-methoxyphenyl)acrylamide (**3**) (DMSO-*d*6, 200 MHz).

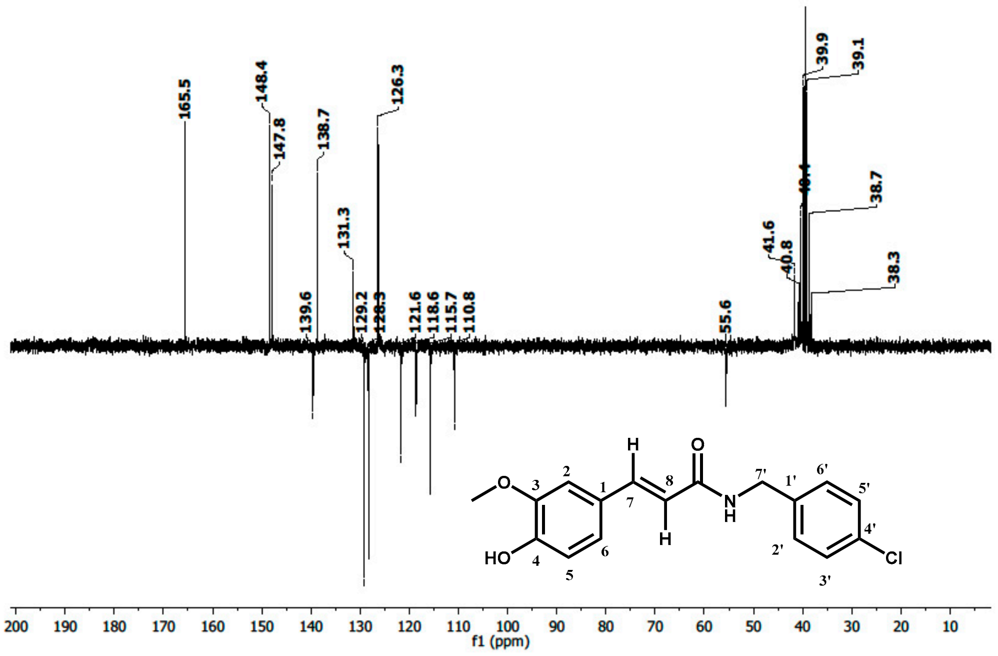


**Figure S10.** $^{13}$C-APT NMR spectrum of (*E*)-*N*-(4-chlorobenzyl)-3-(4-hydroxy-3-methoxyphenyl) acrylamide (**3**) (DMSO-*d*6, 50 MHz).

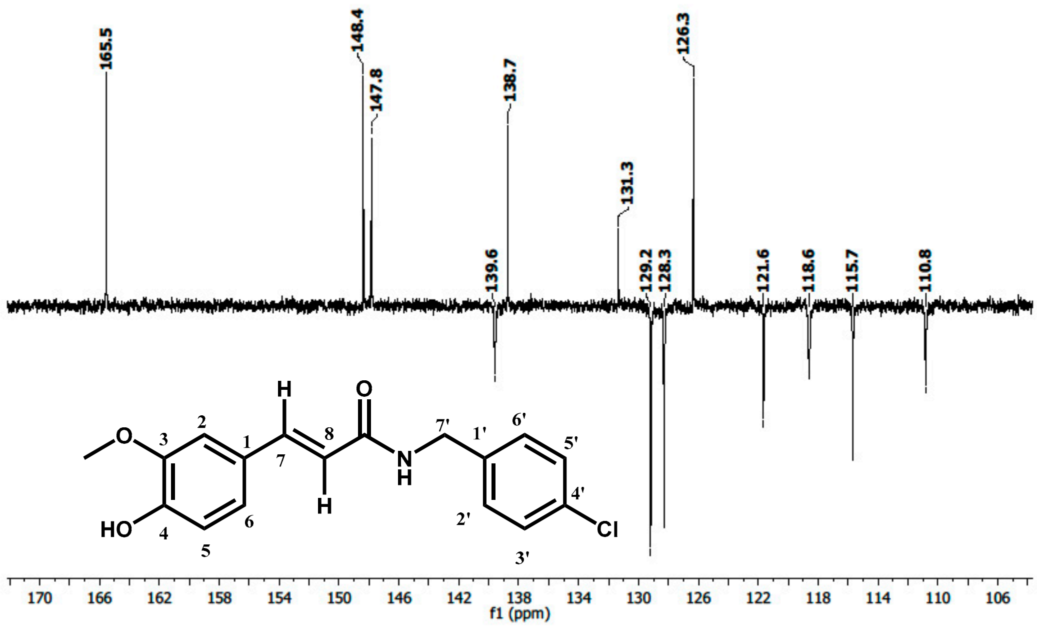


**Figure S11.** Expansion of the ¹³C-APT NMR spectrum of (*E*)-*N*-(4-chlorobenzyl)-3-(4-hydroxy-3-methoxyphenyl)acrylamide (**3**) (DMSO-*d*₆, 50 MHz).

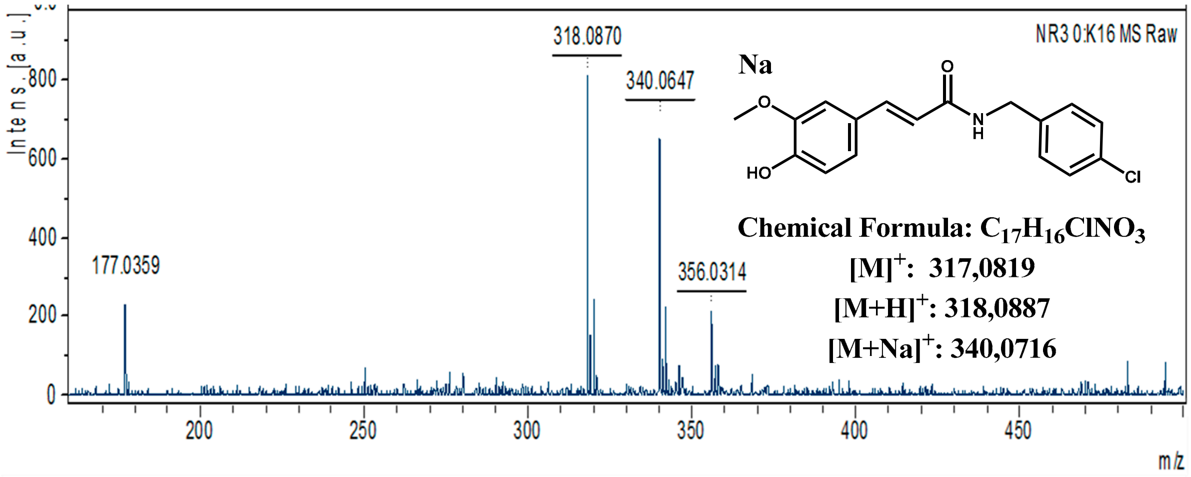


**Figure S12.** High resolution mass spectrum—MALDI of (*E*)-*N*-(4-chlorobenzyl)-3-(4-hydroxy-3-methoxyphenyl) (**3**).

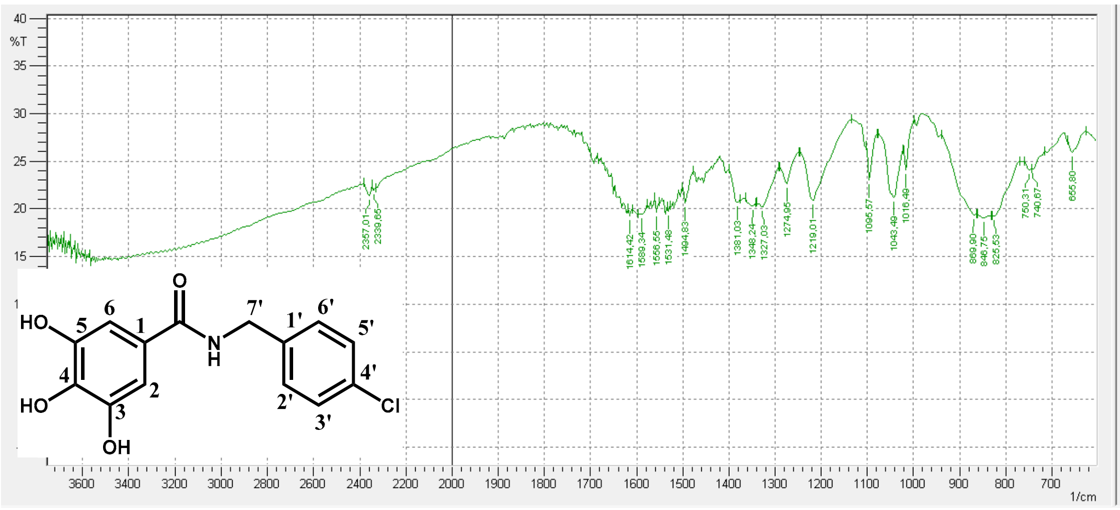

**Figure S13.** Infrared spectrum (KBr, cm$^{-1}$) de *N*-(4-chlorobenzyl)-3,4,5-trihydroxybenzamide (**14**).

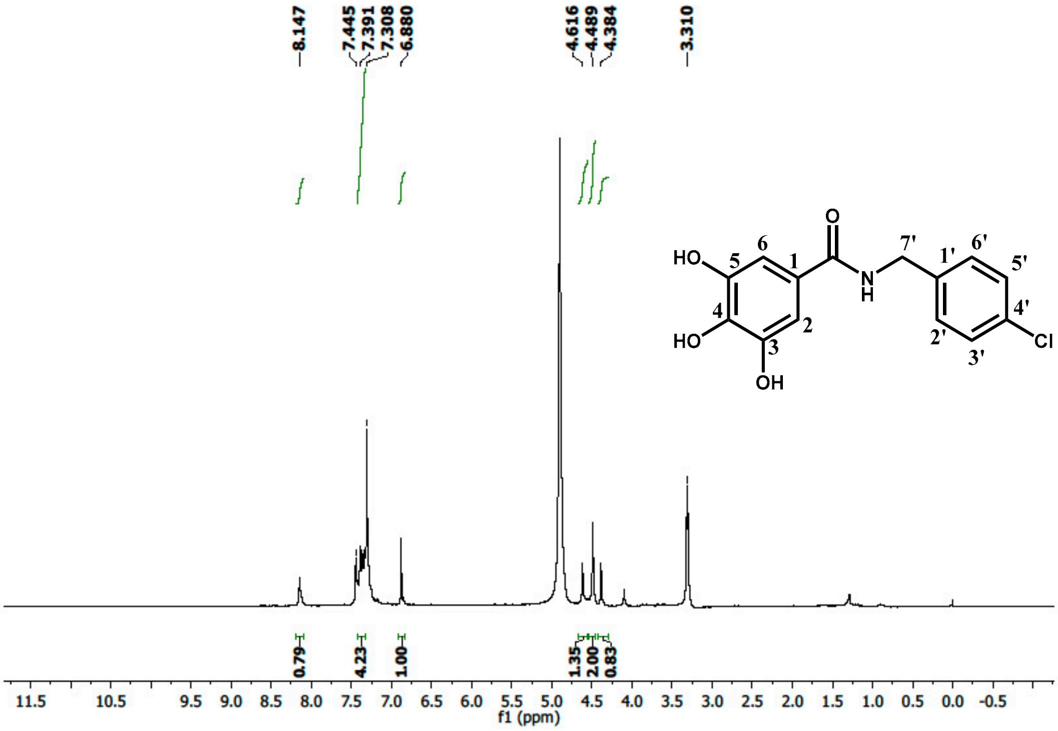

**Figure S14.** $^{1}$H-NMR spectrum of *N*-(4-chlorobenzyl)-3,4,5-trihydroxybenzamide (**14**) (MeOD, 200 MHz).

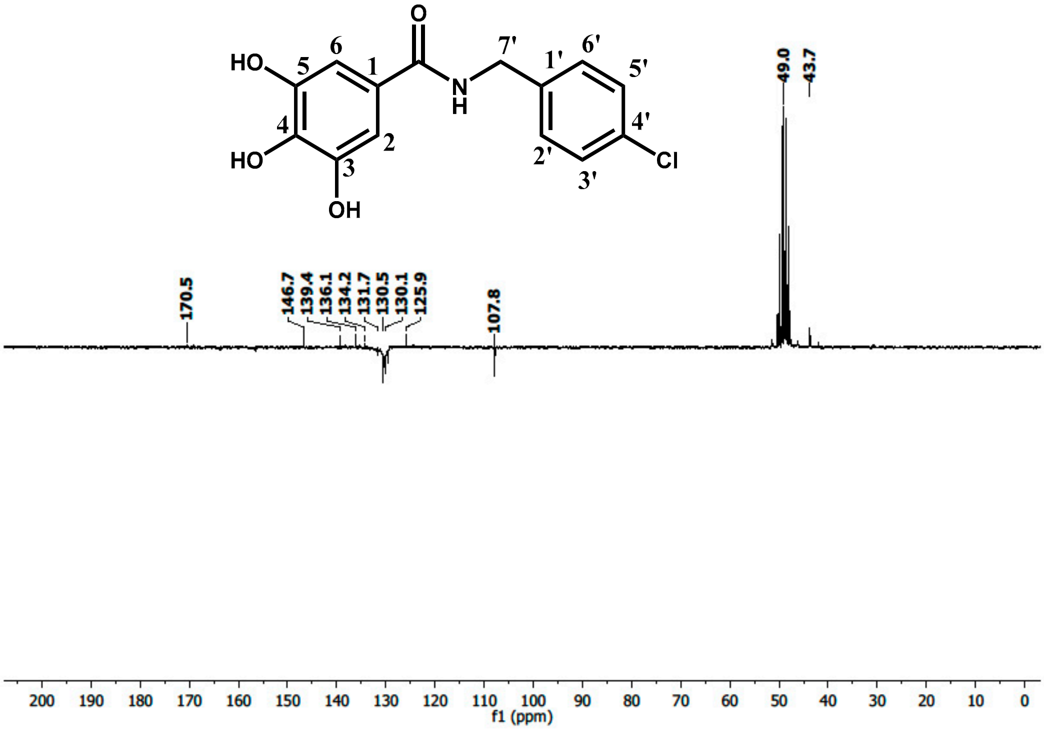


**Figure S15.** $^{13}$C-APT NMR spectrum of *N*-(4-chlorobenzyl)-3,4,5-trihydroxybenzamide (**14**) (MeOD, 50 MHz).

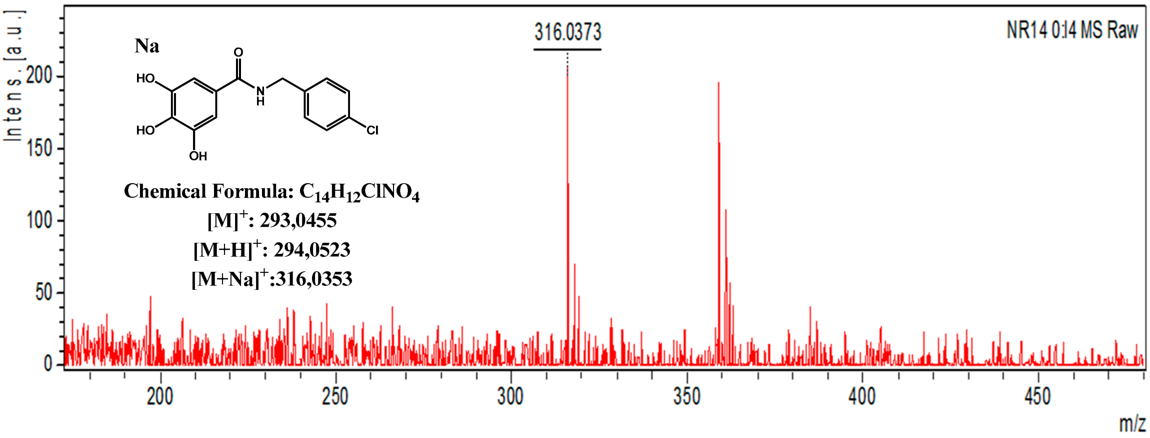


**Figure S16.** High resolution mass spectrum—MALDI of *N*-(4-chlorobenzyl)-3,4,5-trihydroxybenzamide (**14**).

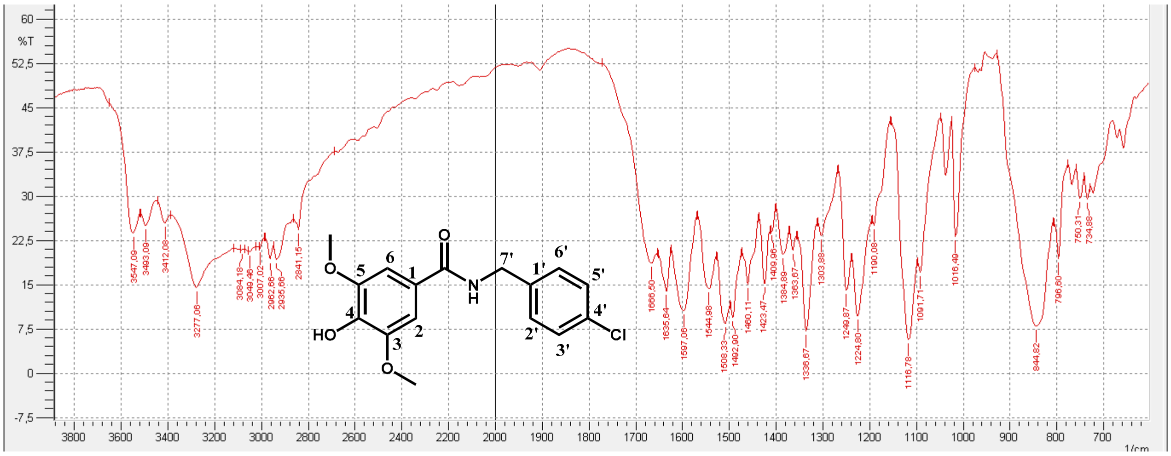

**Figure S17.** Infrared spectrum (KBr, cm$^{-1}$) of *N*-(4-chlorobenzyl)-4-hydroxy-3,5-dimethoxybenzamide (**16**).

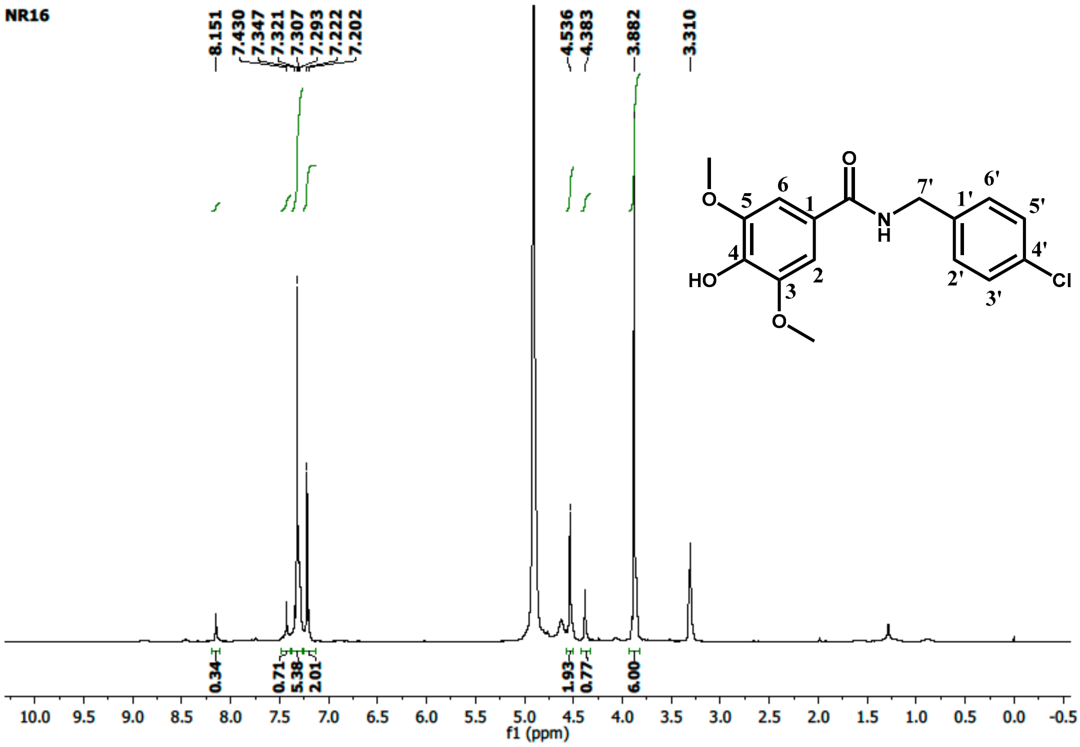

**Figure S18.** $^{1}$H-NMR spectrum of *N*-(4-chlorobenzyl)-4-hydroxy-3,5-dimethoxybenzamide (**16**) (DMSO-$d_6$, 200 MHz).

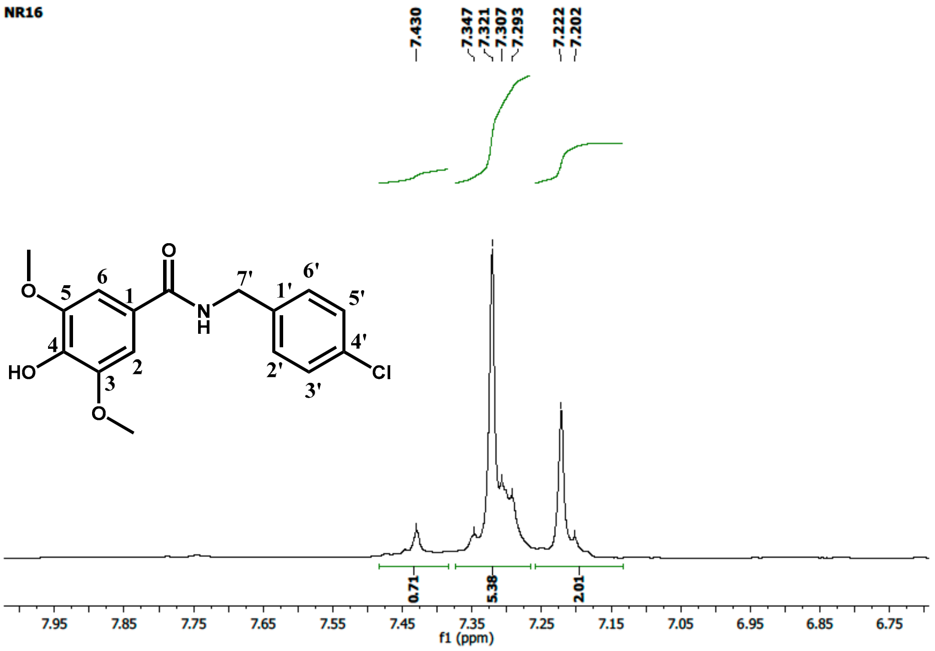


**Figure S19.** Expansion of the $^{1}$H-NMR spectrum of *N*-(4-chlorobenzyl)-4-hydroxy-3,5-dimethoxybenzamide (**16**) (DMSO-$d_6$, 200 MHz).

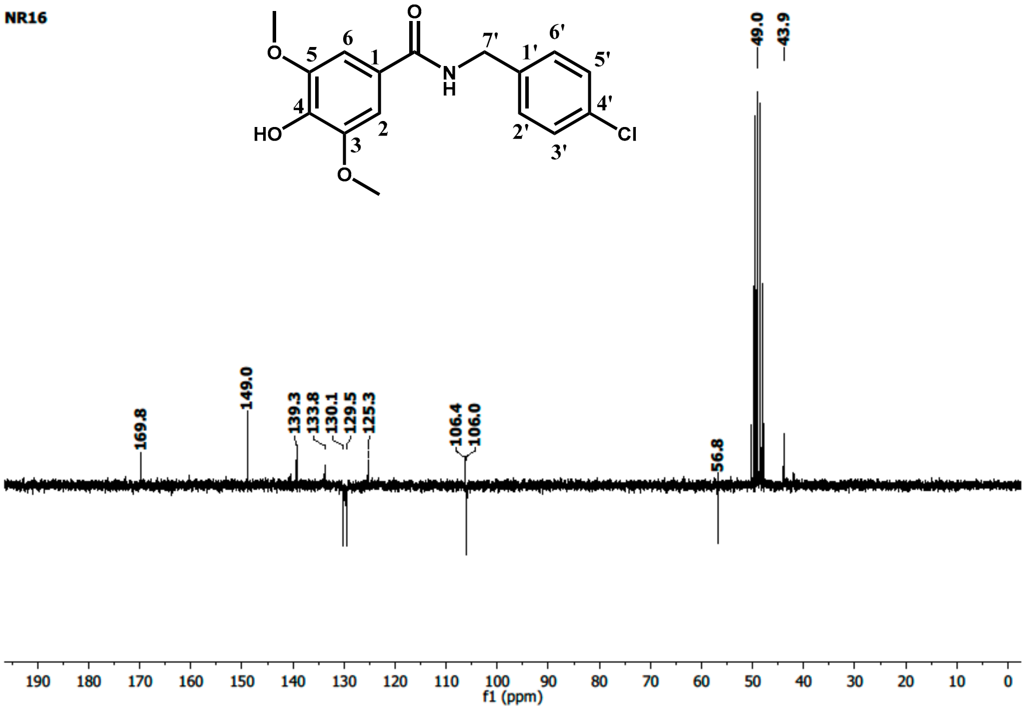


**Figure S20.** $^{13}$C-APT NMR spectrum of *N*-(4-chlorobenzyl)-4-hydroxy-3,5-dimethoxybenzamide (**16**) (DMSO-$d_6$, 50 MHz).

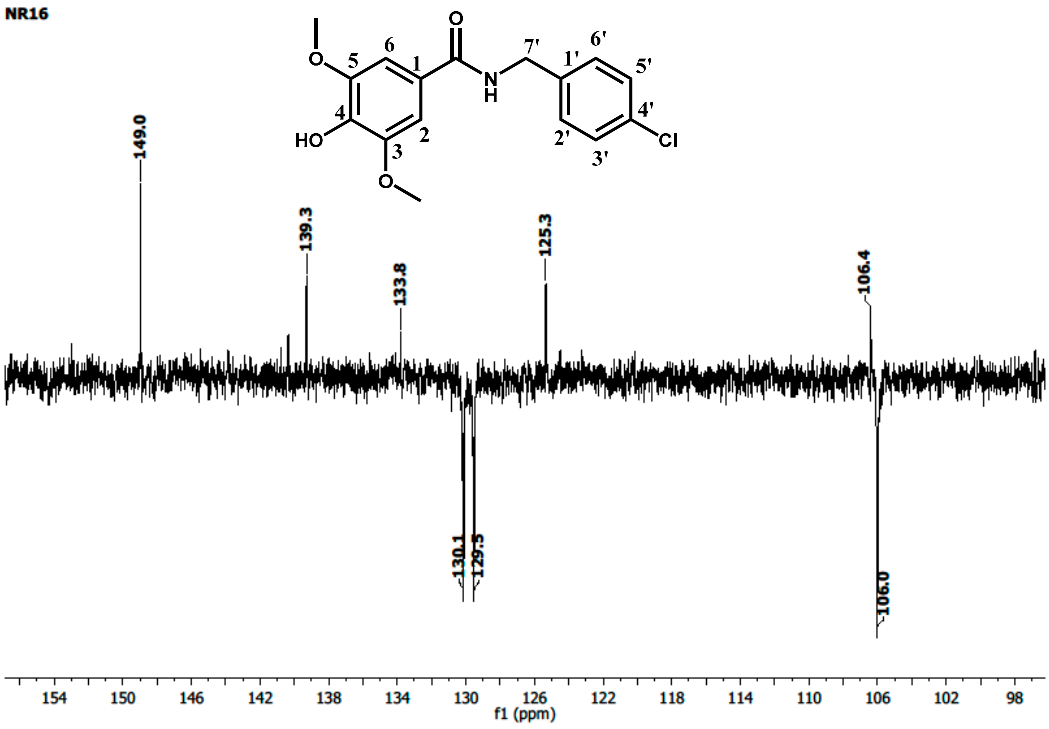


**Figure S21.** Expansion of the spectrum of $^{13}$C-APT NMR of *N*-(4-chlorobenzyl)-4-hydroxy-3,5-dimethoxybenzamide (**16**) (DMSO-*d*6, 50 MHz).

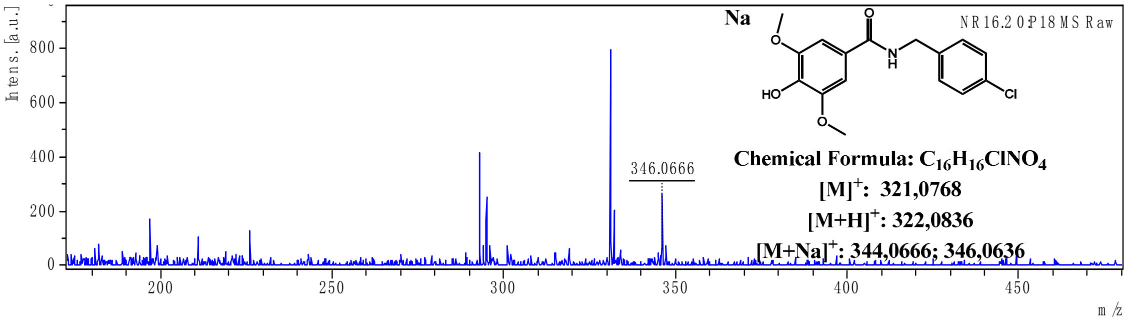


**Figure S22.** High resolution mass spectrum—MALDI of *N*-(4-chlorobenzyl)-4-hydroxy-3,5-dimethoxybenzamide (**16**).

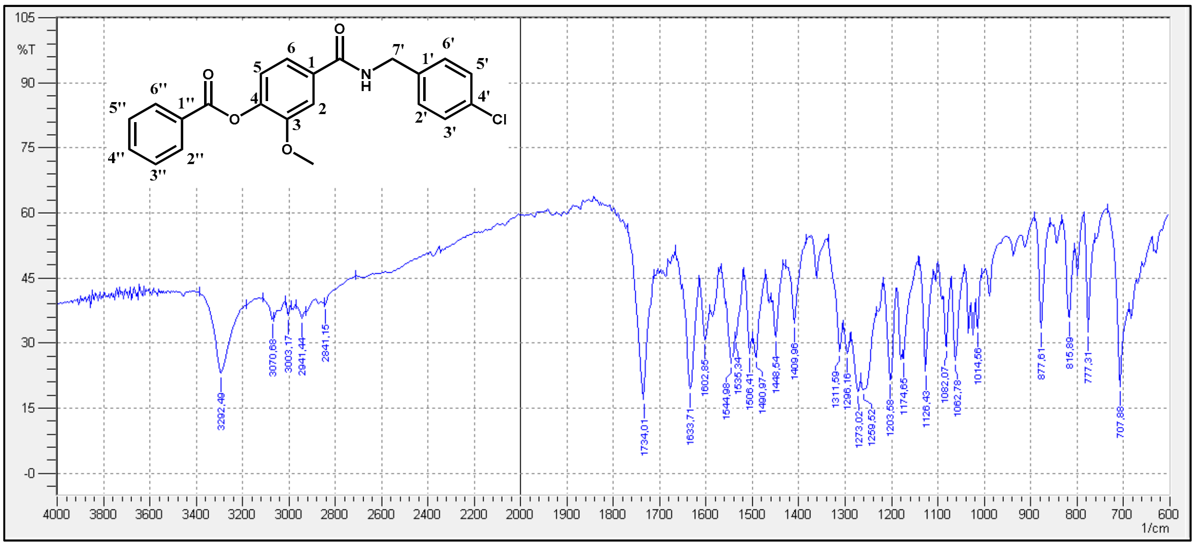

**Figure S23.** Infrared spectrum (KBr, $cm^{-1}$) of 4-((4-chlorobenzyl) carbamoyl)-2-methoxyphenyl benzoate (**23**).

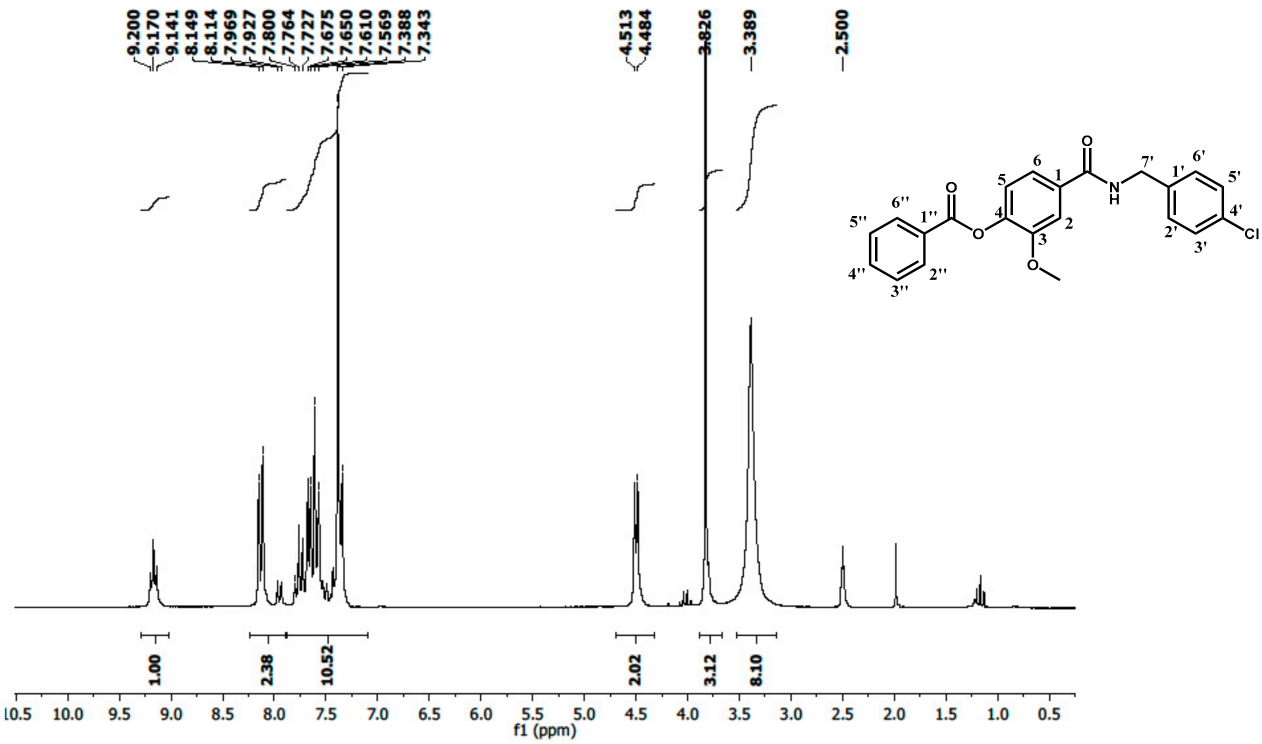

**Figure S24.** $^{1}$H-NMR spectrum of 4-((4-chlorobenzyl) carbamoyl)-2-methoxyphenyl benzoate (**23**) (DMSO-$d_6$, 200 MHz).

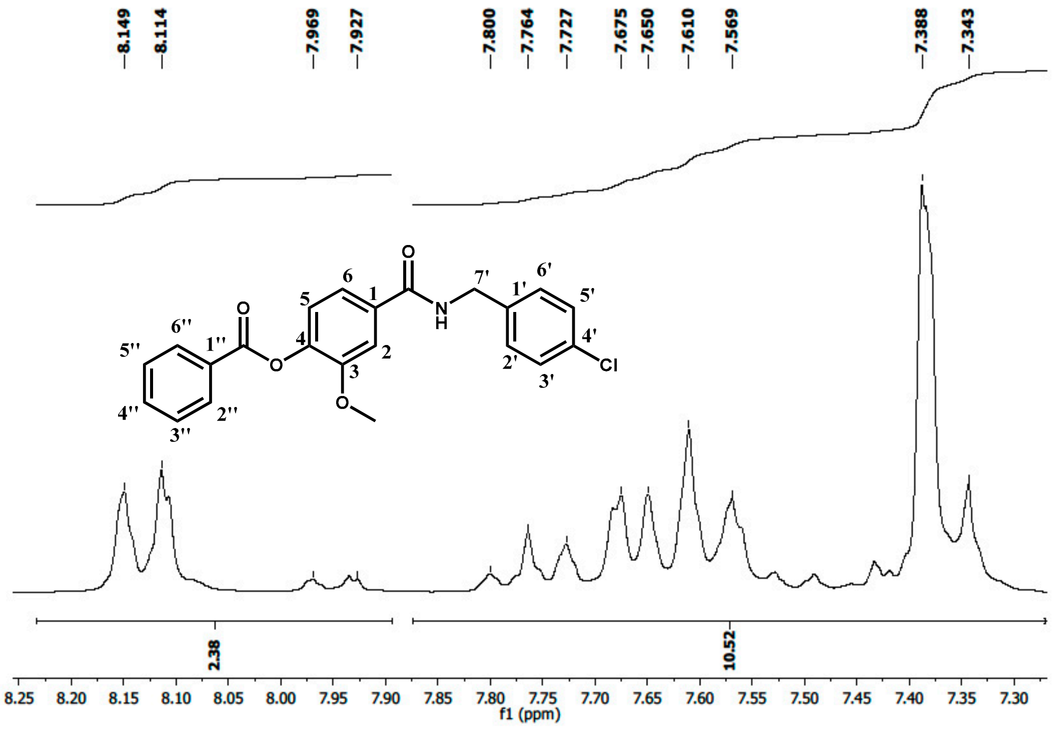


**Figure S25.** Expansion of the $^{1}$H-NMR spectrum of 4-((4-chlorobenzyl) carbamoyl)-2-methoxyphenyl benzoate (**23**) (DMSO-$d_6$, 200 MHz).

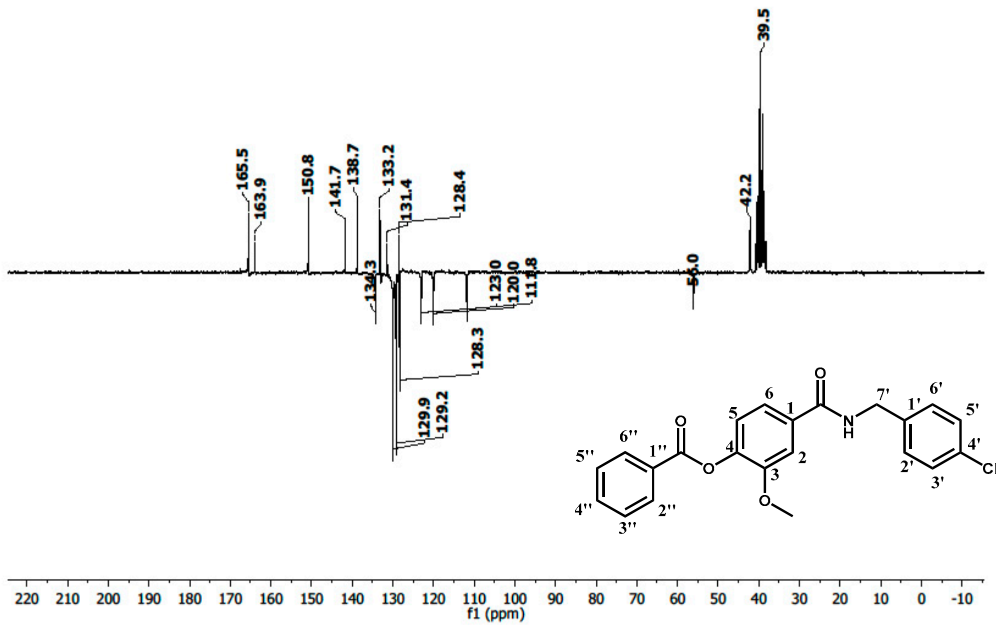


**Figure S26.** $^{13}$C-APT NMR spectrum of 4-((4-chlorobenzyl) carbamoyl)-2-methoxyphenyl benzoate (**23**) (DMSO-$d_6$, 50 MHz).

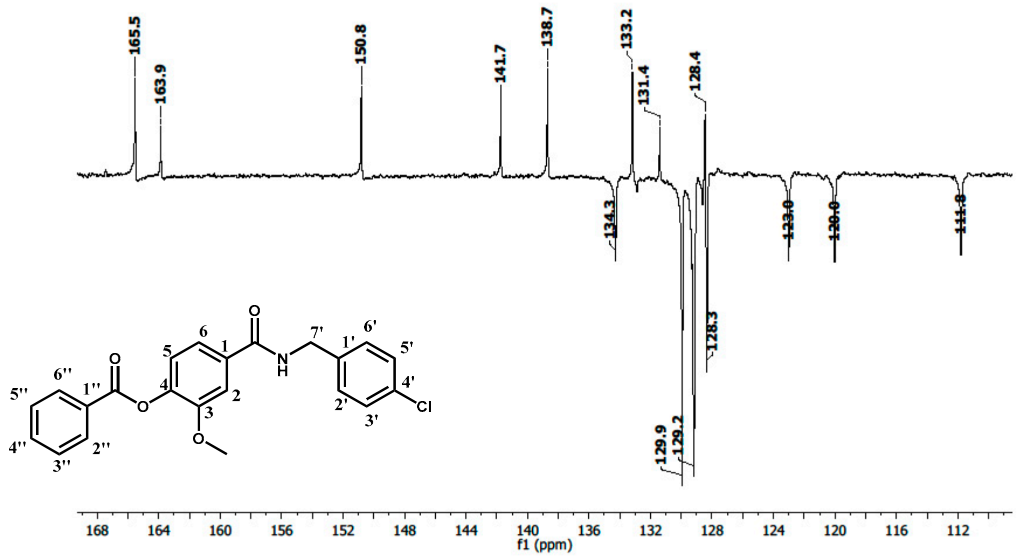


**Figure S27.** Expansion of the $^{13}$C-APT NMR spectrum of 4-((4-chlorobenzyl) carbamoyl)-2-methoxyphenyl benzoate (**23**) (DMSO-*d*$_6$, 50 MHz).

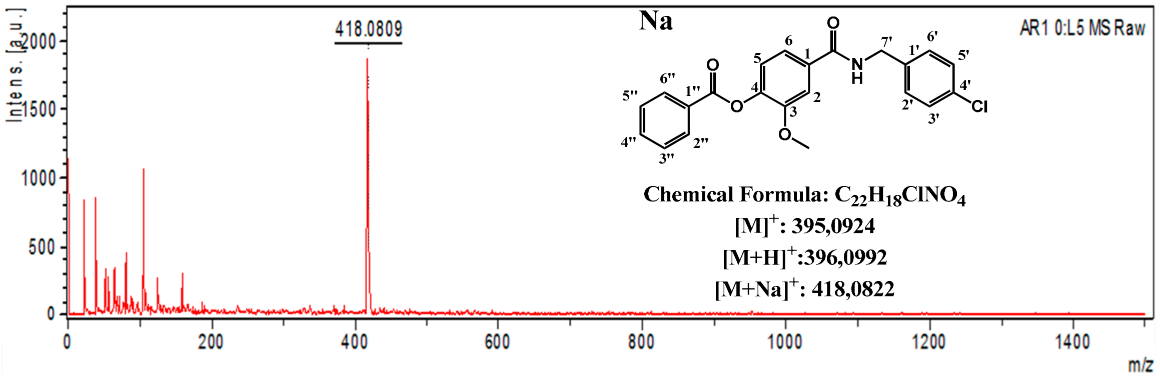


**Figure S28.** High resolution mass spectrum—MALDI of 4-((4-chlorobenzyl)carbamoyl)-2-methoxyphenyl benzoate (**23**).

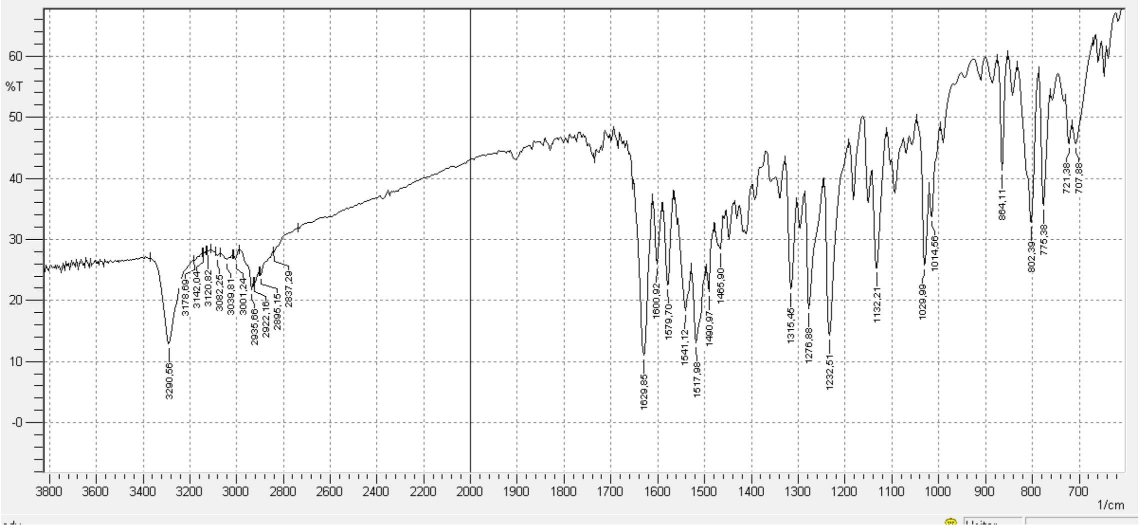


**Figure S29.** Infrared spectrum (KBr, cm$^{-1}$) of *N*-(4-chlorobenzyl)-3-methoxy-4-(4-methylphenetoxy) (**28**).

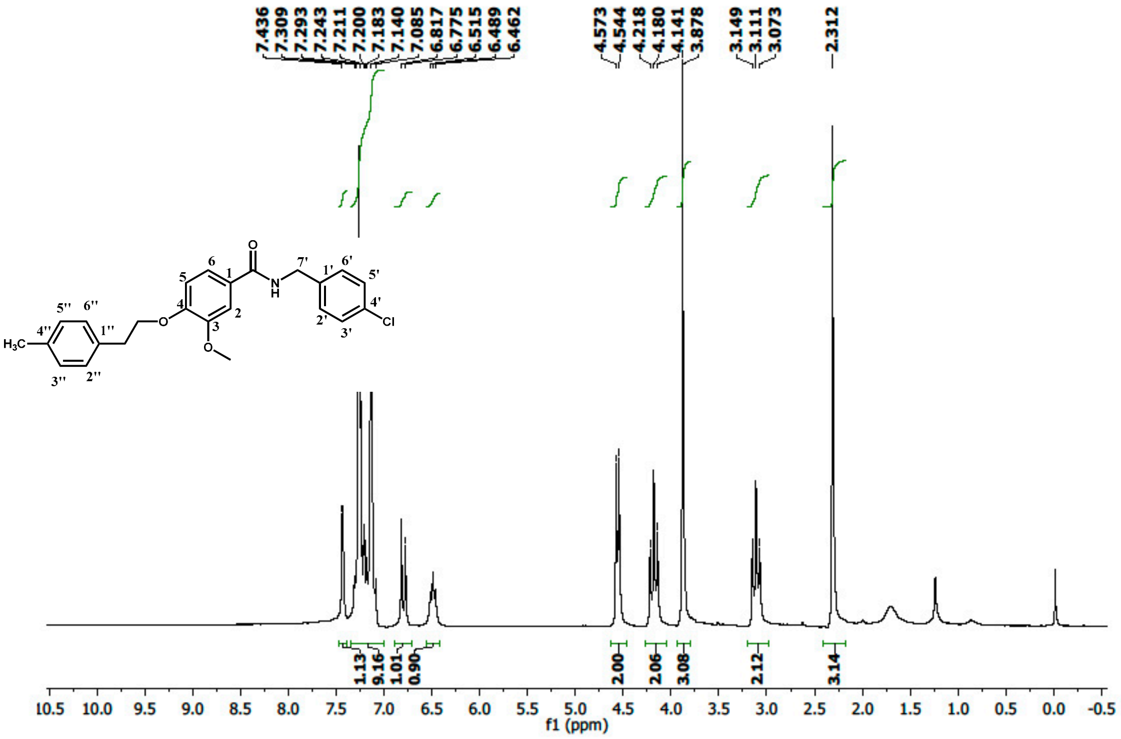


**Figure S30.** $^1$H-NMR spectrum of *N*-(4-chlorobenzyl)-3-methoxy-4-(4-methylphenetoxy) (**28**) ($CDCl_3$, 200 MHz).

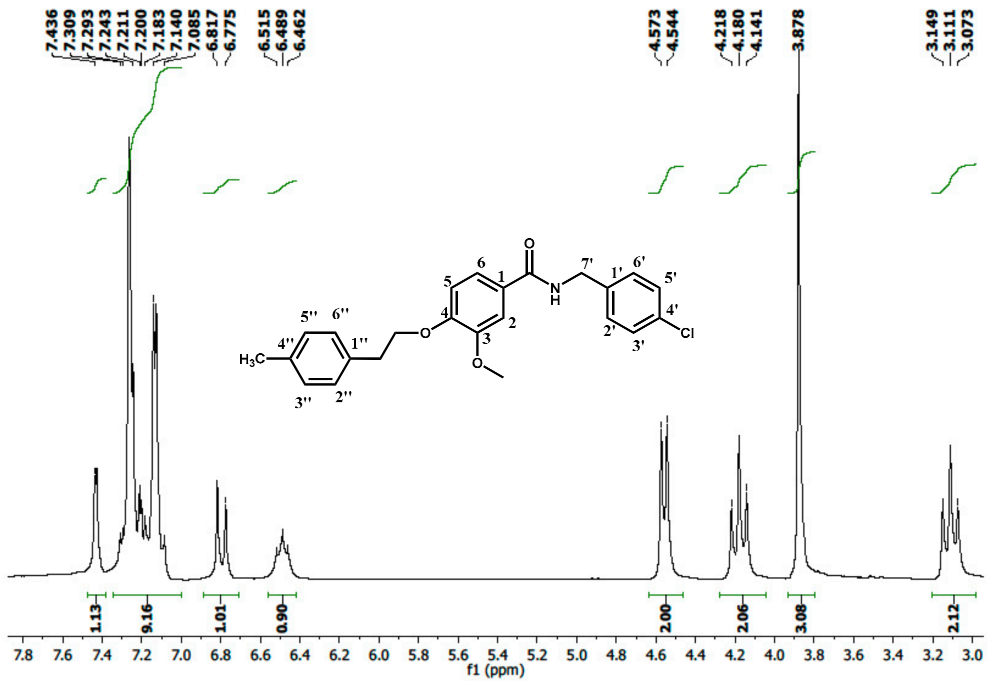


**Figure S31.** Expansion of the spectrum of $^{1}$H NMR of *N*-(4-chlorobenzyl)-3-methoxy-4-(4-methylphenetoxy) (**28**) ($CDCl_3$, 200 MHz).

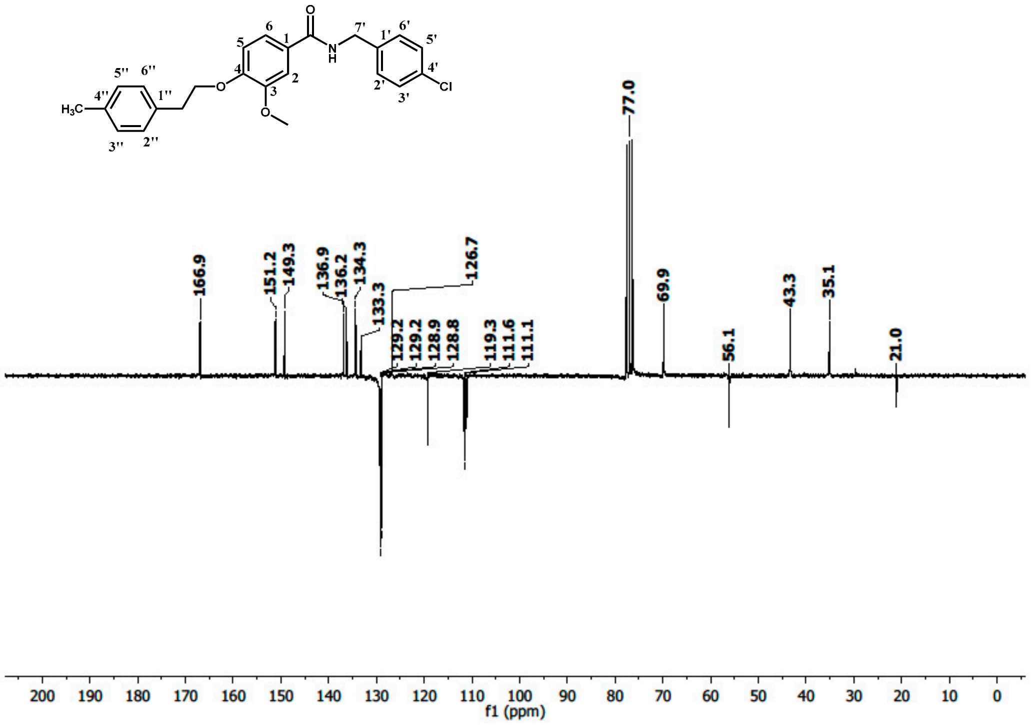


**Figure S32.** $^{13}$C-APT NMR spectrum of *N*-(4-chlorobenzyl)-3-methoxy-4-(4-methylphenetoxy) (**28**) ($CDCl_3$, 50 MHz).

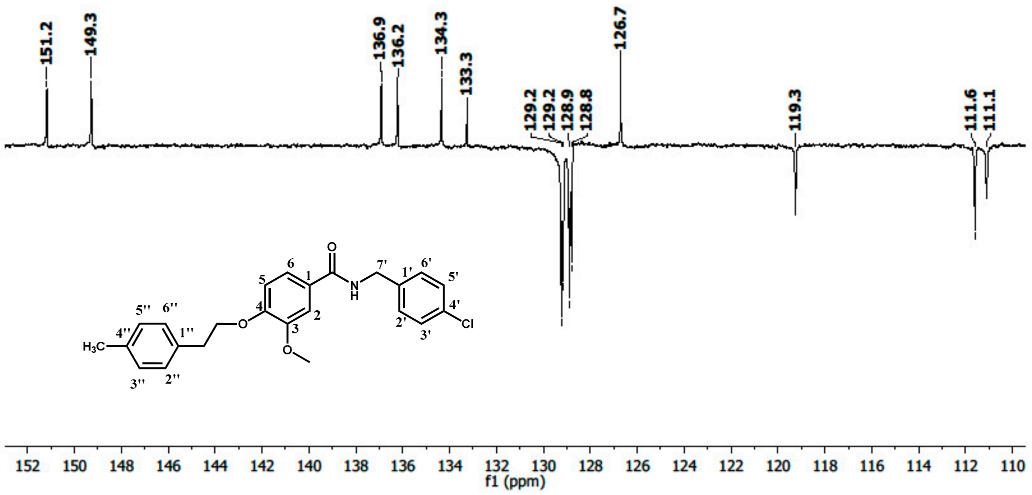


**Figure S33.** Expansion of the $^{13}$C-APT NMR spectrum of *N*-(4-chlorobenzyl)-3-methoxy-4-(4-methylphenetoxy) (**28**) ($CDCl_3$, 50 MHz).

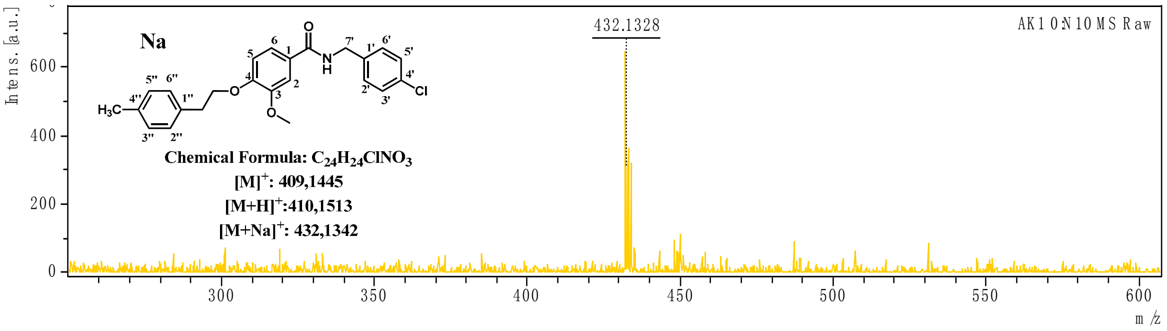


**Figure S34.** High resolution mass spectrum—MALDI of *N*-(4-chlorobenzyl)-3-methoxy-4-(4-methylphenetoxy) (**28**).
